# Laminated self-standing covalent organic framework membrane with uniformly distributed subnanopores for ionic and molecular sieving

**DOI:** 10.1038/s41467-019-14056-7

**Published:** 2020-01-30

**Authors:** Yang Li, Qianxun Wu, Xinghua Guo, Meicheng Zhang, Bin Chen, Guanyi Wei, Xing Li, Xiaofeng Li, Shoujian Li, Lijian Ma

**Affiliations:** 1 Key Laboratory of Radiation Physics and Technology, Ministry of Education, College of Chemistry, Sichuan University, No. 29 Wangjiang Road, Chengdu, 610064 P. R. China; 2 Sichuan University-Pittsburgh Institute, Sichuan University, Chengdu, 610207 P. R. China; 3 Institute of Nuclear Physics and Radiochemistry, Northwest Institute of Nuclear Technology, Xi’an, 710024 P. R. China

**Keywords:** Polymers, Chemical engineering, Organic molecules in materials science

## Abstract

The preparation of subnanoporous covalent-organic-framework (COF) membranes with high performance for ion/molecule sieving still remains a great challenge. In addition to the difficulties in fabricating large-area COF membranes, the main reason is that the pore size of 2D COFs is much larger than that of most gas molecules and/or ions. It is urgently required to further narrow their pore sizes to meet different separation demands. Herein, we report a simple and scalable way to grow large-area, pliable, free-standing COF membranes via a one-step route at organic–organic interface. The pore sizes of the membranes can be adjusted from >1 nm to sub-nm scale by changing the stacking mode of COF layers from AA to AB stacking. The obtained AB stacking COF membrane composed of highly-ordered nanoflakes is demonstrated to have narrow aperture (∼0.6 nm), uniform pore distribution and shows good potential in organic solvent nanofiltration, water treatment and gas separation.

## Abstract (web summary)

Fabrication of large scale and defect free covalent organic framework (COF) membranes with pores small enough for gas sieving remains challenging. Here, the authors report a scalable fabrication method to grow large area defect free COF membranes and to tune the pore size in the sub-nm region by adjusting the stacking modes of the COF layers.

## Introduction

2D covalent organic frameworks (COFs) are layered organic crystalline polymers material with graphite-like topological structure. Based on abundant monomers, flexible bonding modes, and reversible covalent bonding, 2D COFs have the advantages of predesignable structure, tunable pore size, uniform pore distribution, and skeletal diversity^1–5^. These features make 2D COF a promising candidate for high quality membrane materials^6–8^. In addition, unlike other polymer and graphene membranes, additional physical or chemical methods will not be employed for creating pores or changing interlayer space in COF membrane preparation, and there is generally no need for postfunctionalization to adjust pore aperture, which actually simplifies the preparation process^9,10^.

In early studies, it is technically difficult to directly obtain the desired pristine membrane because the methods, such as solvothermal method, microwave method, etc. used for COF synthesis mainly generate stacked or 3D bulk powders^11–14^. Despite some attempts have been made on free-standing or supported COF membranes, progress is very limited^6–8,15,16^. So researchers tried to fabricate COF membranes by vacuum filtration with the experience from preparation of graphene membranes. But before preparing membranes by vacuum filtration, ultrasonic peeling, mechanical grinding, and/or other methods are often required to peel the bulk COF materials into 2D COF nanosheets. However in this process, usually only a tiny mass fraction of the nanosheets can be obtained, and it is difficult to control the size distribution and thickness of the nanosheets^12–14^. Thus, most of the COF membranes prepared through vacuum filtration would craze after drying. As a further solution, mixed matrix membranes strategy was proposed to fabricate the COF-based composite membrane composed of filler COF particles within continuous polymer matrices^17–22^. In essence, they should be COF-modified polymer membranes. Although the transport performance of these composite membranes to molecules and ions is superior to that of pristine polymer membranes^18^, the researchers are not satisfied and continue to explore new ways to prepare COF nanofiltration membranes.

In recent years, interface-confined reactions have been employed to make pristine COF membranes for the purpose of better control of the membrane structure and clearer understanding of the structure–performance relationship. Some researchers successfully got the pristine membranes from gas–solid^23–28^, gas–liquid^29–32^, and water–organic interfaces^29,33^, respectively. Among the three interfacial reaction methods, the water–organic system is regarded as a more ideal platform for preparing 2D COF materials due to relatively mild preparation conditions and much easier separation of the products from the reaction mixture. However, the fact that most of COF monomers have low or no solubility in water limits the range of monomers available for the above interface-confined systems, which affects the extension of current methodologies.

In 2017, Banerjee and colleagues^33^ reported a pioneering method for fabrication of large-scale thin COF membranes under ambient conditions by water–organic interfacial polymerization. In this study, the aldehyde organic linker was dissolved in dichloromethane, while the amine monomers were dissolved in water. To increase the solubility of amine monomers, amines were firstly salt-mediated by p-toluene sulfonic acid (PTSA). Besides increasing the solubility of amine, PTSA can also act as the catalyst during the organic Schiff base reaction. More recently, a Lewis-acid-catalyzed interfacial polymerization approach at water–organic interface by using Sc(OTf)_3_ was developed^34^. Different from the traditional water–organic interfacial polymerization, the Lewis-acid catalyst was preferentially dissolved in water, while the aldehyde monomers (PDA) and amine monomers (TAPB) were dissolved in an organic phase. Surprisingly, the polymerization did not proceed homogeneously in the organic phase, but occur site selectively at the interface of the two phases to provide a polymer membrane.

Although the solubility problem confronted by traditional water–organic interfacial polymerization for COF membrane preparation can be partially or all solved by the above two methods, so far the pore sizes (>1 nm) of the obtained COF membranes are usually much larger than small molecules (H_2_, N_2_, CO_2_, etc.) and ions, which limits their applications in selectively molecule sieving and desalination, etc. Therefore, for the specific separation requirements, it is urgent to further narrow the pore sizes of the membranes to sub-nm scale. However, as far as size-dependent separation with COF membrane is concerned, it has been proved to be very difficult to accurately control the pore sizes in the sub-nm scale, either through control of monomer size or through pore modification^6–8^. Recently, Yang et al.^35^ reported several COF membranes with sub-nm pores prepared by vacuum filtration using 2D COF nanosheets and 1D cellulose nanofibers (CNFs) as starting materials. Due to the shielding effect of CNFs, the aperture of obtained COF-based membrane is in the range of 0.45–1.0 nm. However, a major problem with this method is that the acquisition of subnanopores is at the expense of pore uniformity. Significantly, this work inspired us that if we could use the sheltering effect of the 2D COF layer itself rather than 1D CNF or another COF membrane, we could also obtain a sub-nm pore, but with much more uniform pore distribution. In 2018, Fan et al.^36^ developed a method for preparing COF membranes with narrowed apertures by the formation of interlaced pore networks in a COF–COF bilayer membrane. We think the narrowed apertures can also be obtained in only one COF by control or adjust the stacking mode of two adjacent layers. So, the question becomes how could we control or adjust the stacking mode of two adjacent COF layers? In 2018, a study reported by Cui and colleagues^37^ revealed that the stacking mode and chemical stability of 2D COFs could be controlled via steric tuning. By cocondensation of aldehyde and triamines with and without alkyl substituents (ethyl and isopropyl), they found that, as the steric resistance increases, the stacking mode of COF layers could transform from AA to AB or ABC. So, we wonder if the stacking mode of COF layers could also be adjusted by merely changing the number of functional groups on COF monomers, for the reason that the steric resistance increases as the number of functional groups increases to some extent.

**Fig. 1:**
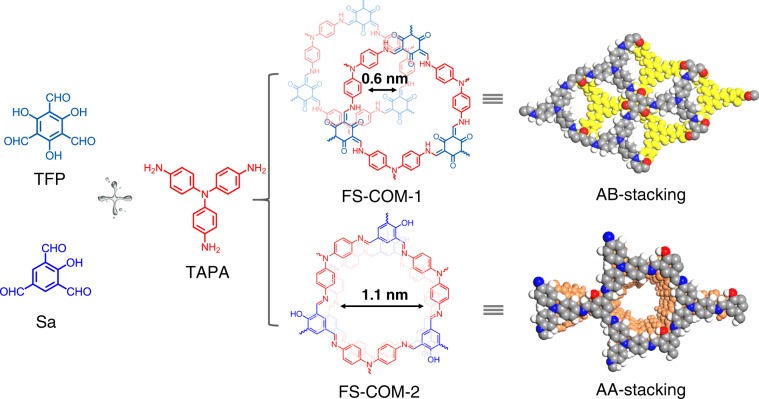
Structures of the monomers and the as-designed two free-standing COF membranes. TFP, Sa, and TAPA is short for 1,3,5-triformylphloroglucinol, 2,4,6-triformylphenol, and tris(4-aminophenyl)amine, respectively. The red, blue, gray, and white spheres represent O, N, C, and H atoms, respectively.

Herein, we report a simple and scalable way to grow a large-area, pliable, and free-standing COF membrane via a modified interfacial polymerization method. In this case, two miscible organic solvents that have good solubility to almost all COF monomers are chosen to solve the above-mentioned solubility problem. Whereafter, a low density solvent is used as a buffer interlayer between the two miscible solvents to stave off the mutual dissolution and maintain the interface between the two main solvents. Meanwhile, in order to obtain subnanoporous COF membrane, we tried to simply increase the number of phenolic hydroxyl groups on triformylbenzene monomers for adjusting the stacking mode of 2D COF layers from AA stacking to AB stacking mode, so as to reduce the pore size from >1 nm to sub-nm scale (Fig. 1). Subsequently, two resulting laminated free-standing covalent-organic-framework membranes (FS-COM-1 and FS-COM-2) with different pore sizes are successfully synthesized and systematically studied, and their selective separation performance for molecules and ions are investigated and also compared.

## Results

### Characterizations of FS-COM-1 and FS-COM-2

**Fig. 2:**
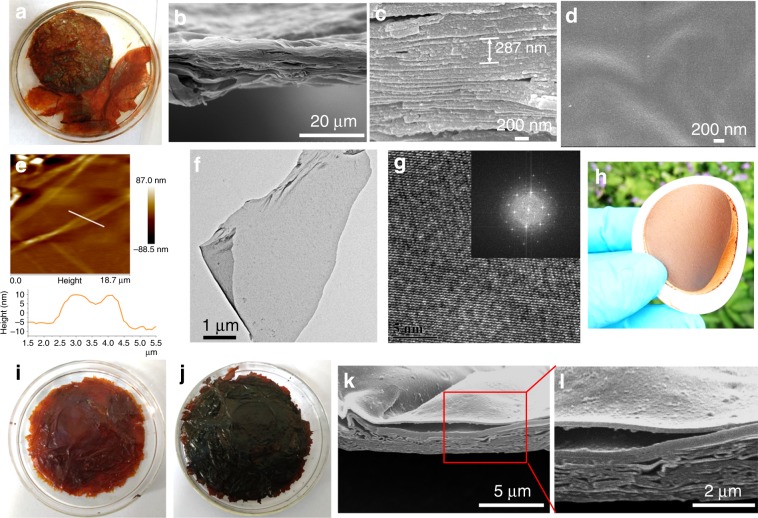
Morphology characterizations. **a** Pristine FS-COM-1 collected from the organic–organic solvent interface. **b**, **c** Cross-section SEM images of FS-COM-1. **d** Top-view SEM image of FS-COM-1. **e** AFM image and the thickness of the nanosheet. **f** TEM image of the nanosheet with micrometer scale. **g** High-resolution TEM patterns. **h** Digital images of substrate-supported membrane (FS-COM-1-VF). **i** Pristine FS-COM-2 collected from the organic–organic solvent interface (soaked in acetone). **j** Pristine FS-COM-2 collected from the organic–organic solvent interface (soaked in water). **k**, **l** Cross-section SEM images of FS-COM-2. Enlarged view of the inserted red box in **k** is shown in **l**.

FS-COM-1 collected from the organic–organic interface is shown in Fig. 2a. The diameter of the membrane is estimated up to several centimeters (~6 cm), which is proportional to the size of the vessel used (250 mL beaker). Moreover, the layer-by-layer nanosheets could be peeled off easily, even with a spoon. A video about the peeling process is attached in the Supplementary Movie 1. The SEM images in Fig. 2b, c exhibit that FS-COM-1 consists of tens of thinner nanosheets, stacked on top of each other with a total thickness of 10–20 μm (monolayer thickness of ∼20 nm). Figure 2d shows that the surface of the membrane looks pretty smooth even when magnified 30,000 times. Even more gratifying is the fact that FS-COM-1 can directly be installed on a filter for the following molecule/ion sieving experiments. Moreover, the FS-COM-1 could be easily exfoliated into COF nanosheets after ultrasound treatment. The SEM images of the obtained COF nanosheets are shown in Supplementary Fig. 1. The element distribution was examined by energy dispersive X-ray spectroscopy mapping, as shown in Supplementary Fig. 2. It can be seen that the distributions of C/N/O in the material are uniform. AFM image of Fig. 2e reveals that the thickness of the nanosheet is around 20 nm, which is similar to the thickness given by the SEM cross-sectional view (Fig. 2c). The few-layer COF sheet in Fig. 2f looks somewhat like graphene sheet. High-resolution TEM patterns of the nanosheets in Fig. 2g and Supplementary Fig. 3 demonstrate the well-ordered stacking of the layers. The layer distance is about 0.35 nm in relation to the thickness of each layer (Supplementary Fig. 3), which is consistent with a π–π stacking distance of 0.37 nm predicted by Materials Studio simulation (Supplementary Fig. 4). The suspensions of the nanosheets could then be used for the preparation of another pure COF membrane by using vacuum filtration (named as FS-COM-1-VF), as shown in Fig. 2h and Supplementary Fig. 5. FS-COM-1-VF has relatively better mechanical strength due to the stronger π–π interaction between interlayers of the membrane. Thus, the newly-prepared membrane could spontaneously detach from the substrate after drying (Supplementary Fig. 5). The SEM patterns of Supplementary Fig. 6 reveal that FS-COM-1-VF membrane looks much loose compared with the FS-COM-1 membrane collected directly from the organic–organic interface (Fig. 2b, c). Pristine FS-COM-2 nanosheets soaked in acetone and water are shown in Fig. 2i, j, respectively. As we can see, obvious color change was found when the membrane was soaked in acetone and water. We then tried other solvent systems and found that the color of FS-COM-2 nanosheets is brown in common organic solvents, while black in water (Supplementary Fig. 7). The color changes during the repeated solvent exchange follow the same rules. A proposed mechanism of the solvent-triggered chromism of FS-COM-2 is shown in Supplementary Fig. 8. We believe the color change is mainly related to the keto–enol tautomerism in different solvents^38^. The cross-section SEM images of FS-COM-2 are shown in Fig. 2k, l. As we can see, similar to FS-COM-1, it is also a laminated structure. However, the surface of FS-COM-2 is not as smooth as FS-COM-1 (Fig. 2d, l).

**Fig. 3:**
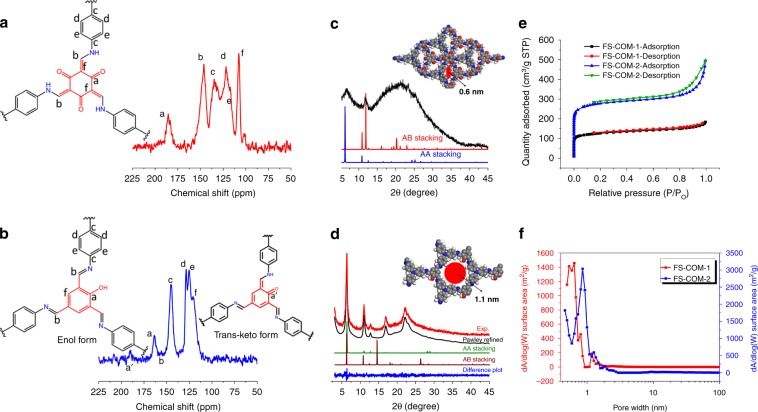
Structural characterizations. 13C solid-state NMR spectra of FS-COM-1 (**a**) and FS-COM-2 (**b**). **c** PXRD patterns of FS-COM-1 comparison between the experimental and the simulated patterns for eclipsed AA stacking mode and AB stacking mode. **d** PXRD patterns of FS-COM-2 comparison between the experimental and Pawley refined profiles, the simulated patterns for eclipsed AA stacking mode, AB stacking mode, and the refinement differences plot. **e** N2 adsorption isotherms of FS-COM-1 and FS-COM-2. **f** Pore size distribution of FS-COM-1 and FS-COM-2. The red, blue, gray, and white spheres represent O, N, C, and H atoms, respectively.

The FT-IR spectrum of FS-COM-1 shows that the characteristic absorption peaks of –NH_2_ (∼3300 cm^−1^) and –CHO (∼2880 and ∼1650 cm^−1^) disappeared after the condensation reaction, while a new peak located at ∼1300 cm^−1^ appeared, which could be attributed to the formation of –C=C–N (Supplementary Fig. 9)^13^. The results could provide preliminary support for the successful formation of objective FS-COM-1 frameworks. Similar conclusions can also be drawn from the FT-IR spectra of FS-COM-2 and the organic monomers (Supplementary Fig. 10). ^13^C CP-MAS solid-state NMR spectroscopy was also employed to verify the atomic-level constructions of the obtained two COF membranes. The results are shown in Fig. 3a, b. In the ^13^C solid-state NMR spectrum of FS-COM-1, the signal located at ∼182 ppm can be ascribed to the formation of C=O of the keto form derived by tautomerism during the reaction process. And the signal at ~146 ppm confirms the presence of the C=C–N bond, which is also in accordance with the keto form structure. Moreover, the absence of C=N (~160 ppm) signal in the spectrum of FS-COM-1 reveals that no enol form structure exists in the framework^13^. The typical XPS survey spectra show that FS-COM-1 is mainly composed of C/N/O (Supplementary Fig. 11a). In the C1s signal (Supplementary Fig. 11b), it can be understood that there are three different carbon environments which could be ascribed to C=C or C–C (∼284.7 eV), C–N (∼286.0 eV), and C=O (∼288.9 eV) respectively^39^. In contrast, the peaks of N1s and O1s are much simpler (Supplementary Fig. 11c, d), which are consistent with the structure of the as-designed FS-COF-1. It is found out that the percentage of O is higher than that of the calculated value (Supplementary Table 1), which may be attributed to the adsorbed water and is also in accordance with the water peak in O1s signal (Supplementary Fig. 11d). The elemental analysis (EA) results show that FS-COM-1 is consisted of four elements (C/H/O/N), and the contents of each elements are close to corresponding calculated values (Supplementary Table 2). The thermogravimetric experiment was carried out under nitrogen protection. As shown in Supplementary Fig. 12, the structure of FS-COM-1 is basically stable within 300 °C and the weight loss before 100 °C is due to the loss of adsorbed water. Furthermore, FS-COM-1 samples were soaked in several different pH solutions for 72 h to test the acid stability. FT-IR spectra in Supplementary Fig. 13 shows that FS-COM-1 is stable in the pH range of 0–7. Unlike FS-COM-1, FS-COM-2 mainly exists in the enol form and only a minority is in the keto form, seen from the NMR spectrum (Fig. 3b). This is supported by the major peak at 163.8 ppm and the minor peak at 189.1 ppm, which can be ascribed to C–OH and C=O of the keto–enol tautomer, respectively^38^. More characterizations of FS-COM-2, such as EA, thermogravimetric analysis, are shown in Supplementary Information (Supplementary Table 3 and Supplementary Fig. 14).

The crystalline structures of FS-COM-1 and FS-COM-2 were confirmed by PXRD and the structural formulae were built in the MS suite of programs. For FS-COM-1, the experimental PXRD pattern is poor, and we are not sure whether FS-COM-1 is AA stacking or AB stacking mode based only on the PXRD (Fig. 3c). We tried a lot to improve the crystal quality by changing the conditions of the interfacial polymerization method, but all failed. We also employed traditional solvothermal method for preparing COFs with high quality XRD. The results are shown in Supplementary Figs. 15–17. As we can see, the PXRD of the resulting COF linked by TFP and TAPA is significantly worse than that of their analogues (Supplementary Figs. 15–17), which are consistent with results reported by Dichtel et al. that beta-ketoenamine-linked COFs typically exhibit smaller average crystalline domain sizes and lower surface areas and pore volumes than imine-linked networks^40^. However, if we focus our attention on the peaks at 15–30°, the simulated PXRD pattern of AB stacking mode matches slightly better with the experimental data than that of AA stacking mode (Fig. 3c). The architectural rigidity and the permanent porosity of FS-COM-1 are further confirmed by N_2_ adsorption–desorption experiments at 77 K to determine its stacking mode. As shown in Fig. 3e, FS-COM-1 presents a typical type-I reversible isotherm, and the adsorption amount in the low pressure region increases rapidly, which is characteristic for a microporous structure^11,41^. The BET surface area and t-Plot micropore area of FS-COM-1 are estimated to be 478 and 431 m² g^−1^, respectively, with the total pore volume of 0.28 cm^3^ g^−1^. Importantly, the narrow pore size distribution of FS-COM-1 with an average pore width of 0.6 nm (Fig. 3f), calculated by the nonlocal density functional theory method, agrees well with the predicted value of AB stacking model (Fig. 3c, Supplementary Figs. 18 and 19). As a contrast, FS-COM-2 shows a much higher quality PXRD (Fig. 3d). And the experimental PXRD pattern agrees well with the pattern simulated from an AA-eclipsed layer stacking model. Pawley refinement against experimental PXRD data provided good agreement factors (*R*wp = 5.70% and *R*p = 4.50%). The permanent porosities of FS-COM-2 were also investigated by nitrogen sorption analysis at 77 K (Fig. 3e). The BET surface area and t-Plot micropore area of FS-COM-2 are estimated to be 1062 and 966 m² g^−1^, respectively, with the total pore volume of 0.76 cm^3^ g^−1^. The pore size distribution of FS-COM-2 exhibited maxima of 0.95 nm, which is in line with the predicted value of AA-eclipsed geometries of the frameworks (Fig. 3d). From PXRD and nitrogen sorption analysis, we can see that the stacking modes of FS-COM-1 and FS-COM-2 were quite different due to the different number of phenolic hydroxyl groups between the two aldehyde monomers (TFP and Sa, see Fig. 1). This is in consistent with our initial design that the stacking modes of 2D COFs can be adjusted by simply changing the number of functional groups of COF monomers.

### Growth process of FS-COM-1 and FS-COM-2

**Fig. 4:**
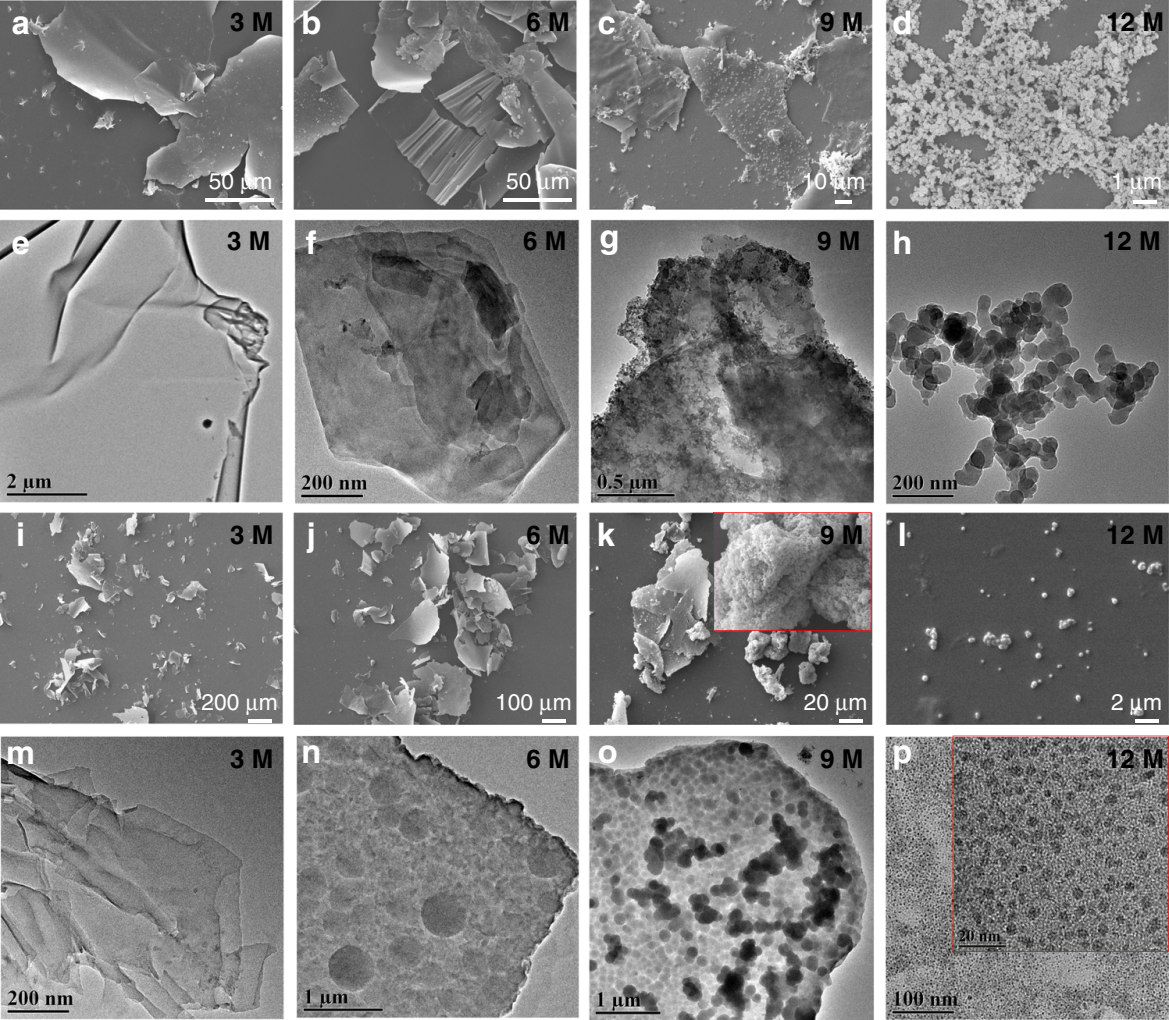
SEM and TEM images of COFs obtained at different acetic acid concentrations. **a**–**d** SEM images of FS-COM-1; **e**–**h** TEM images of FS-COM-1; **i**–**l** SEM images of FS-COM-2; **m**–**p** TEM images of FS-COM-2. Enlarged views of **k** and **p** are shown in the inserted red boxes.

To shed light on the growth process of the laminated self-standing FS-COM-1 and FS-COM-2, various experiments were done at different synthesis conditions. In our case, we think the acetic acid is critical for the membrane formation as there is no obvious interface formation between the two main miscible organic solvents without acetic acid. So, firstly, concentrations (3–12 M) and amounts of acetic acid (20–60 mL) were employed to explore their effects on the experimental results. Not surprisingly, we found that the concentrations of acetic acid are important for the formation of COF membranes. For low acetic acid concentrations of 3 and 6 M, only membranes were obtained in the 250 mL beakers. When the concentration of acetic acid increased to 9 M, a mixture of COF membrane and nanoparticles could be obtained. And as the concentration increased further to 12 M, only COF nanoparticles are generated. After ultrasonic treatment, the resulting products were characterized by SEM and TEM. As we can see in Fig. 4, a continuous morphology changes from nanosheets to nanoparticles or quantum dots can be found with the increasing concentrations of acetic acid from 3 to 12 M, which is in consistent with their initial mother structures. Digital photos of nanosheets and quantum dots of FS-COM-2 are shown in Supplementary Fig. 20. As we can see, the dispersion liquid of COF quantum dots shows the obvious Tyndall effect. In addition to the above experimental results, we also found that the flatness of the COF nanosheets changed with the increasing concentrations of acetic acid, especially for FS-COM-2 (Fig. 4). As we can see from Fig. 4n, o, the nanosheet structures are obviously formed by the accumulation of spherical particles. This may give a hint to the growth process of the COF membranes. Are the laminated self-standing FS-COM-1 and FS-COM-2 membranes formed by the orderly accumulation of COF nanoparticles? To prove this, we then analyzed in detail the FS-COM-1 membrane grown under 9 M acetic acid. Digital photos of top (left) and bottom (right) surfaces of the FS-COM-1 membrane are shown in Supplementary Fig. 21. From the comparison of the two surfaces, it can be seen that the bottom surface, which is in contact with dichloromethane, is much smoother than that of the top one. We further characterized the morphology of the upper, middle, and lower parts of FS-COM-1 grown under 9 M acetic acid by SEM after ultrasonic treatment. The results are shown in Supplementary Fig. 22. As we can see, the surface of the nanosheet is smoother in the lower part of FS-COM-1. Combined with this finding, we proposed a possible growth process for the laminated self-standing membrane. It is worth noting that all membranes are prepared in a 250 mL beaker. The optical image of the reaction system is shown in Supplementary Fig. 23a. As we can see, from top to bottom, three distinct solvent layers can be found in the beaker, which are separately ascribed to the amine solution, acetic acid solution, and aldehyde solution. In the initial stage, amines in DMF passed through the acetic acid layer to meet aldehyde and then formed a yellow COF film at the acetic acid–dichloromethane interface. After the formation of COF film, the diffusion of amines to aldehyde solution was largely hindered for the size of the amine is relatively larger than the pore size of the resulting COF. Moreover, the amine monomers can react with acetic acid to form salt which also make it more difficult for the amines to diffuse into the aldehyde solution. On the contrary, the diffusion of aldehyde monomers to amine layer is much easier for the size of aldehyde is relatively smaller and their diffusion is not affected by acetic acid. Aldehydes that diffused into the amine layer could react with amine rapidly to form COF nanoparticles under the catalysis of acetic acid. Correspondingly, the color of the amine layer was deepened rapidly, while that of the aldehyde layer was basically unchanged during the experiment. The gradually growing COF nanoparticles slowly settled to the COF film initially grown at the acetic acid–dichloromethane interface under the action of gravity, and the film could act as a template to regrow the COF from nanoparticles to another layer of COF film. This process was repeated and finally a laminated self-standing COF membrane was obtained (Supplementary Fig. 23b–g). Then, why does the COF morphology change from membranes to nanoparticles as the concentration of aqueous acetic acid increases? We think it may be related to the initial interface formed by aqueous acetic acid and dichloromethane. The pure acetic acid concentration is about 17.5 M. When the concentration of acetic acid gradually increased to 12 M, most of the components in the solution are acetic acid, the mutual solubility between acetic acid and dichloromethane would affect the formation of the initial acetic acid–dichloromethane interface, and then affect the formation of the initial COF layer, which may eventually lead to a transition of the COF from membranes to nanoparticles. In order to verify the importance of the initial acetic acid–dichloromethane interface, the effect of different acetic acid addition volume (6 M) on the morphology was also studied. With the decreasing amount of acetic acid (60–20 mL), the thickness of acetic acid layer gradually decreases until it disappears. It is worth pointing out that, the volume of acetic acid layer was less than the volume that we added due to the mutual solubility between acetic acid and DMF. The experimental results showed that, as long as there is an acetic acid buffer layer, laminated COF membrane can be obtained. Otherwise, only COF nanoparticles were obtained. PXRD patterns of COF materials obtained at different acetic acid concentrations are shown in Supplementary Figs. 24–26. It can be seen that the PXRD patterns of the resulting COFs also gradually improved with the acetic acid concentration increasing from 3 to 12 M. The results make us confused. If the COF nanosheets are formed by the gradual regrowth of quantum dots, why the PXRD patterns are getting worse? We suspected that there might be a preferred crystal growth orientation. On account of the better crystal quality of FS-COM-2 membrane, we take FS-COM-2 grown under 3 M acetic acid as an example to validate it. The results are shown in Supplementary Fig. 26. It can be seen that PXRD patterns parallel and perpendicular to the membrane surface are quite different. The reflections corresponding to the (100) crystal planes disappear when the X-ray is perpendicular to the membrane surface, which indicates that the surface of the COF membrane may grow along (100) direction. Moreover, the PXRD pattern of the pristine FS-COM-2 membrane is much worse than that of the resulting COF nanosheets after ultrasound treatment. This may be related to the lower density of membrane materials compared with its nanosheet counterparts. Due to the low density of the membrane, we also tried to hit the COF membrane (~0.7 mg) with a laser and to see if it could be driven (The power of the laser pointer is 3 mW). To prevent the influence of air flow, the experiment was carried out in a closed balance (Supplementary Fig. 27). And in the experiment, COF nanosheet and capillary tube were linked by spider silk. Delightedly, the COF membrane can do be driven by the red laser. A video about the light-driven process is attached in the Supplementary Movie 2.

### Membrane performance study

**Fig. 5:**
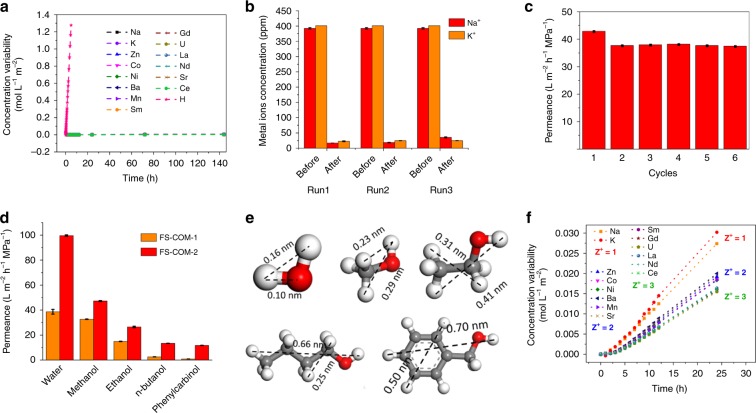
Membrane performance study. **a** Concentration variability of 14 coexisting cations in the permeate part for FS-COM-1. The multi-ion solution containing 14 coexisting cations was prepared by dissolving the desired metal oxides or nitrates in concentrated nitric acid and then diluting them with deionized water. **b** Na_2_SO_4_ or K_2_SO_4_ rejection performance of FS-COM-1 at 0.1 MPa in the three filtration cycles. **c** Water permeance of FS-COM-1 for six filtration cycles. **d** Permeance of solvents with increasing sizes for FS-COM-1 and FS-COM-2. **e** Molecular sizes of the selected solvents calculated by MS. **f** Concentration variability of 14 coexisting cations in the permeate part for FS-COM-1-VF. The error bars in **b** and **d** show standard deviations based on three independent measurements. The red, gray, and white spheres represent O, C, and H atoms, respectively.

We further investigated the ability of the obtained membranes in the applications of water treatment, organic solvent nanofiltration, and gas separation. The ion or dye permeation experiments were conducted using a self-designed U-shaped glass filter, and FS-COM-1, FS-COM-1-VF, or FS-COM-2 membrane is fixed at the joint between the two filter bowls (Supplementary Fig. 28). Rhodamine B molecule, hydrogen ion, and 14 metal ions including mono-, di-, and trivalent forms were chosen to exam the performance of the three FS-COM membranes in filtration experiments. The experimental apparatus and more detailed experimental procedure can be seen in Supplementary Fig. 28 and related part. Our experimental results indicate that all the 14 cations tested could not permeate through FS-COM-1 membrane in ambient conditions, even if the filtration time lasted for 144 h, while H^+^ can easily pass through the membrane (Fig. 5a). Similarly, no penetration of rhodamine B (molecule size 1.7 nm × 1.3 nm, Supplementary Fig. 29) was observed in a 72 h filtration experiment (Supplementary Figs. 30 and 31). In order to test filtration performance of FS-COM-1 under pressure, we also designed and manufactured a pressure filtration apparatus (Supplementary Figs. 32 and 33) for pressure filtration test. The results are shown in Fig. 5b. As we can see, FS-COM-1 exhibited Na_2_SO_4_ or K_2_SO_4_ rejection values between 90 and 95% at 0.1 MPa in the three filtration cycles. We also studied the permeance of common solvents for FS-COM-1 under a transmembrane pressure of 0.1 MPa. The pure water permeation test was repeated 6 times for FS-COM-1 (Fig. 5c), and the average water permeance was validated to be ∼38.6 L m^−2^ h^−1^ MPa^−1^. It is worth pointing out that the permeance of the last five cycles are apparently smaller than that of the first cycle, which may be due to that the membrane is pressed more tightly under pressure. The permeation properties of FS-COM-1 and FS-COM-2 were also studied by passing methanol, ethanol, *n*-butanol, and phenylcarbinol (Fig. 5d), with which the solvent sizes are gradually increasing (Fig. 5e). A direct relationship, for both FS-COM-1 and FS-COM-2, was observed between the membrane permeance and the molecular diameters of the solvents. It can be seen that as the solvent size increases, the permeance becomes smaller. Interestingly, ratio of permeance of water to methanol is 1.1 for FS-COM-1, which is ~2 times smaller than that of FS-COM-2 (2.1). The significant increase in water permeation of FS-COM-2 may be related to the structure change of keto–enol tautomerism in different solvents (Supplementary Figs. 7 and 8). Color changes of FS-COM-2 exposed to the air are shown in Supplementary Fig. 34. The number in the green box is the photo shooting time. As we can see, FS-COM-2 is very easy to absorb water, proving that the material has a good hydrophilic ability. Further analysis of the organic solvent nanofiltration results reveals that, for FS-COM-1, the permeance becomes very slow when the size of solvent is closer to the membrane pore size, and the permeance of phenylcarbinol is only 0.9 L m^−2^ h^−1^ MPa^−1^ which is about 36 times slower than that of methanol. However, for FS-COM-2, the phenylcarbinol permeance is only four times slower than that of methanol (Supplementary Figs. 35 and 36). The apparent size-dependent permeance is related to the difference in pore size between FS-COM-1 and FS-COM-2. We also tried to use FS-COM-1 for membrane separation of noble gases xenon (Xe) and krypton (Kr). Experimental apparatus and detailed experimental conditions are shown in Supplementary Information (Supplementary Fig. 37, Supplementary Table 4). The separation of Xe/Kr has important applications in many fields, such as atmospheric radionuclide monitoring, industrial preparation of noble gases, and spent fuel treatment etc. The conventional method to separate these two gases is fractional distillation at cryogenic temperatures, which is energy intensive and costly. In addition, even after cryogenic distillation, trace levels of radioactive Kr in the Xe-rich phase are too high to permit further use^42^. In principle, membrane separation can well overcome the drawbacks of fractional distillation. Under the experimental conditions, the permeance of Xr and Kr for FS-COM-1 is 1.158 × 10^−8^ and 1.286 × 10^−8^ mol m^−2^ s^−1^ Pa^−1^, and the separation coefficient was calculated to be about 1.12. In order to reduce the test error caused by unbalanced system, we sampled three times every 10 min. The results are shown in Supplementary Table 4. As we can see, the permeance and separation coefficient kept unchanged for three consecutive samples. Besides, the selective metal ion separation performance of FS-COM-2 and FS-COM-1-VR in multi-ion solution were also studied. The experimental results are shown in Fig. 5f and Supplementary Fig. 38. As we can see, all the ions in the experiment can pass though FS-COM-2 and FS-COM-1-VR. Interestingly, FS-COM-1-VR exhibits a distinctive group separation capability for 14 metal ions based on the ionic valences, which might be applied in separating and recovering valuable metals from heavy metal- or radionuclide-contaminated effluents. It is worth noting that although the UO_2_^2+^ ion is formally divalent, its sieving curve is at the bottom of all curves. This could be ascribed to that the apparent charge of UO_2_^2+^ is 3.2+ ^43,44^.

## Discussion

Modern membrane materials, whether used for the traditional separation and purification or newly emerging transportation of ions, molecules and other species, face three bottleneck problems in commercializing process: (1) trade-off between permeability and selectivity, which limits separation efficiency; (2) mechanical, chemical, and thermal stabilities, which limit practical performance; and (3) fabrication technology and cost, which limit commercialization potential. Herein, we successfully fabricated the expected COF membrane (FS-COM-1) at 2D-confined organic–organic interfaces. The pristine membrane exhibits high ion/molecule exclusion ability and moderate water permeability, and thus potential for real applications in desalination and water purification. For almost all of the reported COF membranes, as far as we know, the pore size is above 1 nm. It is not good for improving selectivity of the membranes especially to metal ions, though a larger pore size usually means a faster filtration rate. In addition, the proposed method can address the shortcoming of insufficient solubility of most organic monomers in water–organic system, which extends the application range of the interfacial reaction methods in COF synthesis. Meanwhile, due to the addition of acetic acid as a buffer layer, the confined interface can be widened, which is beneficial to increase the thickness and strength of the membrane, and thus the prepared membrane can be directly used in the membrane separation process. In principle, the sizes of the membranes prepared by this method are proportional to that of the vessel used. Consequently, our study makes a meaningful attempt to overcome the above three bottlenecks.

## Methods

### Synthesis of FS-COM-1 or FS-COM-2

Firstly, a certain amount of 1,3,5-triformylphloroglucinol (TFP, 21 mg) or 2,4,6-triformylphenol (Sa, 17.8 mg) was dissolved in 80 mL dichloromethane to give the aldehyde solution (A); then 3–12 M acetic acid solution was added slowly until the surface of A was completely covered. Amine solution (B) was prepared by dissolving tris(4-aminophenyl)amine (TAPA, 29 mg) in 50 mL DMF. Next, the solution B was added dropwise to the surface of acetic acid solution. After a period of time, the product was collected and washed thoroughly with dichloromethane, ethanol, acetone and DMF in turn, and put aside for further investigation.

### Synthesis of FS-COM-1-VF

FS-COM-1-VF membrane was prepared by using a vacuum-assisted self-assembly technique which was detailed as follows: amount of as-prepared FS-COM-1 was dispersed in 200 mL deionized water and followed by ultrasonic treatment (SCIENTZ-II D, 280 W, 8 h) to form COF suspension. Then, the as-prepared COF colloidal suspension was transferred to a commercial CA–CN membrane to fabricate the substrate-supported membrane, via a vacuum-assisted self-assembly method. The newly-obtained membrane can spontaneously detach from the substrate after drying, which again formed a free-standing COF membrane (donated as FS-COM-1-VF).

## Supplementary information

Supplementary Information

Description of Additional Supplementary Files

Supplementary Movie 1

Supplementary Movie 2

## Data Availability

All data supporting the findings of this study are available within the article, as well as the Supplementary Information file, or available from the corresponding authors on reasonable request.

## Source data

Source Data

## References

[1] Waller PJ, Gandara F, Yaghi OM (2015). Chemistry of covalent organic frameworks. Acc. Chem. Res..

[2] Huang N, Wang P, Jiang D (2016). Covalent organic frameworks: a materials platform for structural and functional designs. Nat. Rev. Mater..

[3] Feng X, Ding X, Jiang D (2012). Covalent organic frameworks. Chem. Soc. Rev..

[4] Diercks CS, Yaghi OM (2017). The atom, the molecule, and the covalent organic framework. Science.

[5] Colson JW, Dichtel WR (2013). Rationally synthesized two-dimensional polymers. Nat. Chem..

[6] Zhang C, Wu BH, Ma MQ, Wang Z, Xu ZK (2019). Ultrathin metal/covalent-organic framework membranes towards ultimate separation. Chem. Soc. Rev..

[7] Yuan S (2019). Covalent organic frameworks for membrane separation. Chem. Soc. Rev..

[8] Wang H (2019). Recent progress in covalent organic framework thin films: fabrications, applications and perspectives. Chem. Soc. Rev..

[9] O’Hern SC (2014). Selective ionic transport through tunable subnanometer pores in single-layer graphene membranes. Nano Lett..

[10] Aghigh A (2015). Recent advances in utilization of graphene for filtration and desalination of water: a review. Desalination.

[11] Rao MR, Fang Y, De Feyter S, Perepichka DF (2017). Conjugated covalent organic frameworks via michael addition-elimination. J. Am. Chem. Soc..

[12] Khayum MA (2016). Chemically delaminated free-standing ultrathin covalent organic nanosheets. Angew. Chem. Int. Ed..

[13] Chandra S (2013). Chemically stable multilayered covalent organic nanosheets from covalent organic frameworks via mechanical delamination. J. Am. Chem. Soc..

[14] Bunck DN, Dichtel WR (2013). Bulk synthesis of exfoliated two-dimensional polymers using hydrazone-linked covalent organic frameworks. J. Am. Chem. Soc..

[15] Shan M (2018). Facile manufacture of porous organic framework membranes for precombustion CO2 capture. Sci. Adv..

[16] Fan H, Gu J, Meng H, Knebel A, Caro J (2018). High-flux membranes based on the covalent organic framework COF-LZU1 for selective dye separation by nanofiltration. Angew. Chem. Int. Ed..

[17] Zou C (2017). Mechanical synthesis of COF nanosheet cluster and its mixed matrix membrane for efficient CO2 removal. ACS Appl. Mater. Interfaces.

[18] Zhu X, Tian C, Do-Thanh CL, Dai S (2017). Two-dimensional materials as prospective scaffolds for mixed-matrix membrane-based CO2 separation. ChemSusChem.

[19] Shan M (2016). Azine-linked covalent organic framework (COF)-based mixed-matrix membranes for CO2 /CH4 separation. Chem. Eur. J..

[20] Kang Z (2016). Mixed matrix membranes (MMMs) comprising exfoliated 2D covalent organic frameworks (COFs) for efficient CO2 separation. Chem. Mater..

[21] Cheng Y, Wang Z, Zhao D (2018). Mixed matrix membranes for natural gas upgrading: current status and opportunities. Ind. Eng. Chem. Res..

[22] Biswal BP, Chaudhari HD, Banerjee R, Kharul UK (2016). Chemically stable covalent organic framework (COF)-polybenzimidazole hybrid membranes: enhanced gas separation through pore modulation. Chem. Eur. J..

[23] Zwaneveld NA (2008). Organized formation of 2D extended covalent organic frameworks at surfaces. J. Am. Chem. Soc..

[24] Xu L (2013). Surface-confined crystalline two-dimensional covalent organic frameworks via on-surface schiff-base coupling. ACS Nano.

[25] Xu L (2014). Surface-confined single-layer covalent organic framework on single-layer graphene grown on copper foil. Angew. Chem. Int. Ed..

[26] Liu XH (2013). On-surface synthesis of single-layered two-dimensional covalent organic frameworks via solid-vapor interface reactions. J. Am. Chem. Soc..

[27] Dienstmaier JF (2012). Isoreticular two-dimensional covalent organic frameworks synthesized by on-surface condensation of diboronic acids. ACS Nano.

[28] Dienstmaier JF (2011). Synthesis of well-ordered COF monolayers: surface growth of nanocrystalline precursors versus direct on-surface polycondensation. ACS Nano.

[29] Sahabudeen H (2016). Wafer-sized multifunctional polyimine-based two-dimensional conjugated polymers with high mechanical stiffness. Nat. Commun..

[30] Payamyar P (2014). Synthesis of a covalent monolayer sheet by photochemical anthracene dimerization at the air/water interface and its mechanical characterization by AFM indentation. Adv. Mater..

[31] Murray DJ (2015). Large area synthesis of a nanoporous two-dimensional polymer at the air/water interface. J. Am. Chem. Soc..

[32] Dai W (2016). Synthesis of a two-dimensional covalent organic monolayer through dynamic imine chemistry at the air/water interface. Angew. Chem. Int. Ed..

[33] Dey K (2017). Selective molecular separation by interfacially crystallized covalent organic framework thin films. J. Am. Chem. Soc..

[34] Matsumoto M (2018). Lewis-acid-catalyzed interfacial polymerization of covalent organic framework films. Chem.

[35] Yang H (2019). Covalent organic framework membranes through a mixed-dimensional assembly for molecular separations. Nat. Commun..

[36] Fan H (2018). Covalent organic framework-covalent organic framework bilayer membranes for highly selective gas separation. J. Am. Chem. Soc..

[37] Wu X, Han X, Liu Y, Liu Y, Cui Y (2018). Control interlayer stacking and chemical stability of two-dimensional covalent organic frameworks via steric tuning. J. Am. Chem. Soc..

[38] Li X (2018). Molecular engineering of bandgaps in covalent organic frameworks. Chem. Mater..

[39] Okpalugo TIT, Papakonstantinou P, Murphy H, McLaughlin J, Brown NMD (2005). High resolution XPS characterization of chemical functionalised MWCNTs and SWCNTs. Carbon.

[40] Daugherty MC (2019). Improved synthesis of beta-ketoenamine-linked covalent organic frameworks via monomer exchange reactions. Chem. Commun..

[41] Sick T (2018). Oriented films of conjugated 2D covalent organic frameworks as photocathodes for water splitting. J. Am. Chem. Soc..

[42] Fernandez CA, Liu J, Thallapally PK, Strachan DM (2012). Switching Kr/Xe selectivity with temperature in a metal-organic framework. J. Am. Chem. Soc..

[43] Wang L (2017). Effective charge-discriminated group separation of metal ions under highly acidic conditions using nanodiamond-pillared graphene oxide membrane. J. Mater. Chem. A.

[44] Choppin GR, Rao LF (1984). Complexation of pentavalent and hexavalent actinides by fluoride. Radiochim. Acta.

