# Supplementary material for: Laminated self-standing covalent organic framework membrane with uniformly distributed subnanopores for ionic and molecular sieving
Source: Nat Commun. 2020 Jan 30;11:599. doi: 10.1038/s41467-019-14056-7 (PMC6992836; doi:10.1038/s41467-019-14056-7)
Supplement: Supplementary file 1 — Supplementary Information [file 41467_2019_14056_MOESM1_ESM.pdf]

Laminated self-standing covalent organic framework membrane with uniformly distributed subnanopores for ionic and molecular sieving

Li et al

Supplementary Methods

Materials

1,3,5-triformylphloroglucinol (TFP) was prepared according to the published procedure.[1] Tris(4-aminophenyl)amine (TAPA), metal oxides and nitrates was purchased from Shanghai Aladdin Biochemical Technology Co.,Ltd. 2,4,6-triformylphenol (Sa) was purchased from Shanghai Kaiyulin Pharmaceutical Technology Co., Ltd. All the commercially available chemicals are used as received without further purification.

Characterization methods

FT-IR spectra were measured by a NEXUS 670 FT-IR spectrometer. ^{13}C NMR spectra were recorded on a Bruker Avance III 400 WB (400 MHz) NMR spectrometer. Element analysis was performed on a CARLO ERBA 1106. XPS measurement was performed with a Kratos ASAM800 spectrometer. TGA were carried out on a DTG-60(H) analyzer under N_2 atmosphere at a heating rate of 10 ºC min^{-1}. PXRD were collected on X'Pert Pro MPD DY129 diffractometer using Cu Kα radiation at 40 kV, 40 mA power. SEM was characterized by a JSM-7500F Scanning Electron Microscope. AFM (Innova) scans were collected at 512 points/lines under tapping mode in air. TEM characterizations were recorded using Tecnai G2 F20 S-TWIN TEM at an accelerating voltage of 300 kV. Metal ion concentrations were detected by ICP-AES using the standard curve method.

Synthetic procedures

FS-COM-1 or FS-COM-2

FS-COM-1 or FS-COM-2 were prepared by a modified buffering interlayer interface (BII) method according to the published procedure.[2] Firstly, a certain amount of TFP (21 mg) or Sa (17.8 mg) was dissolved in 80 mL dichloromethane to give the aldehyde solution (A); then 3-12 M acetic acid solution was added slowly until the surface of A was completely covered. Amine solution (B) was prepared by dissolving 29 mg TAPA in 50 mL DMF. Next, the solution B was added dropwise to the surface of acetic acid solution. After a period of time, the product was collected and washed thoroughly with dichloromethane, ethanol, acetone and DMF in turn, and put aside for further investigation.

FS-COM-1-VF

FS-COM-1-VF membrane was prepared by using a vacuum-assisted self-assembly technique

which was detailed as follows: amount of as-prepared FS-COM-1 was dispersed in 200 mL deionized water and followed by ultrasonic treatment (SCIENTZ-II D, 280 W, 8 h) to form COF suspension. Then, the as-prepared COF colloidal suspension was transferred to a commercial CA-CN membrane to fabricate the substrate-supported membrane, via a vacuum-assisted self-assembly method. The newly-obtained membrane can spontaneously detach from the substrate after drying, which again formed a free-standing COF membrane (donated as FS-COM-1-VF).

Supplementary Figures

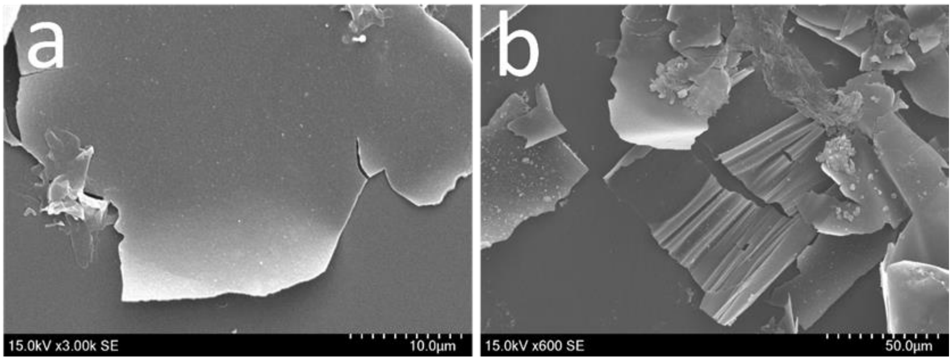

Supplementary Fig. 1 SEM images of FS-COM-1 nanosheets after ultrasonic treatment.

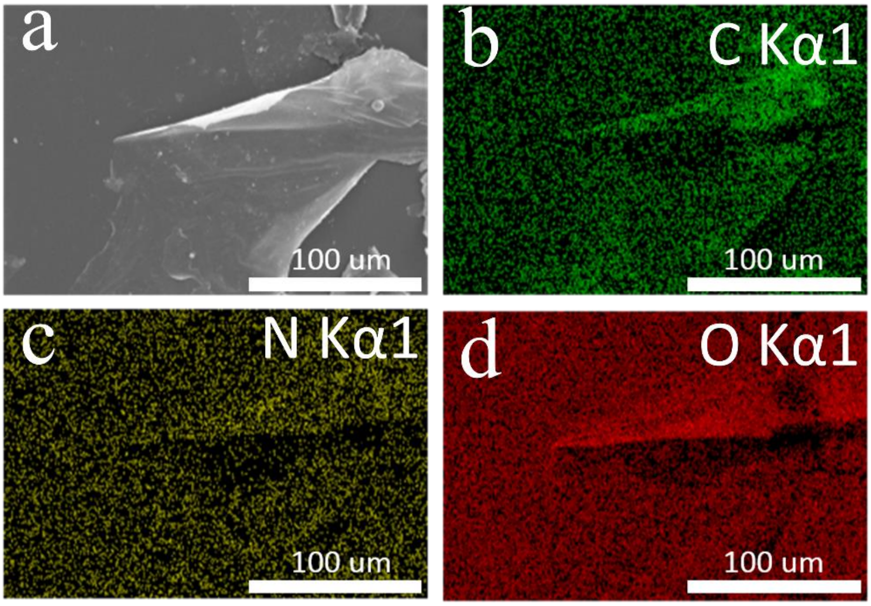

Supplementary Fig. 2 SEM image (a) and element distribution of C (b) / N (c) / O (d) examined by energy dispersive X-ray spectroscopy mapping.

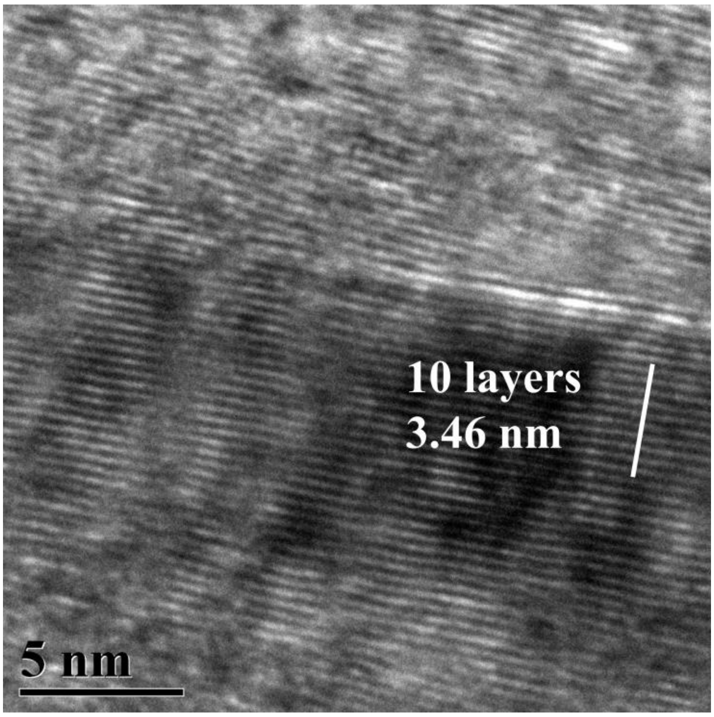

Supplementary Fig. 3 High-resolution TEM patterns of FS-COM-1 after ultrasound treatment.

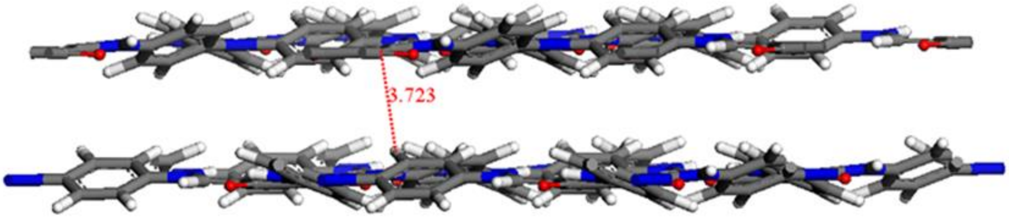

Supplementary Fig. 4 π–π stacking distance (3.7 Å,0.37 nm) for AB stacking model of FS-COM-1 predicted by MS simulation.

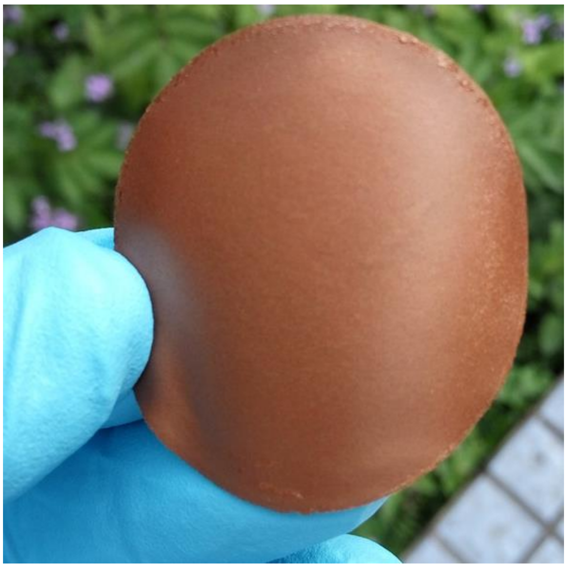

Supplementary Fig. 5 Digital image of FS-COM-1-VF.

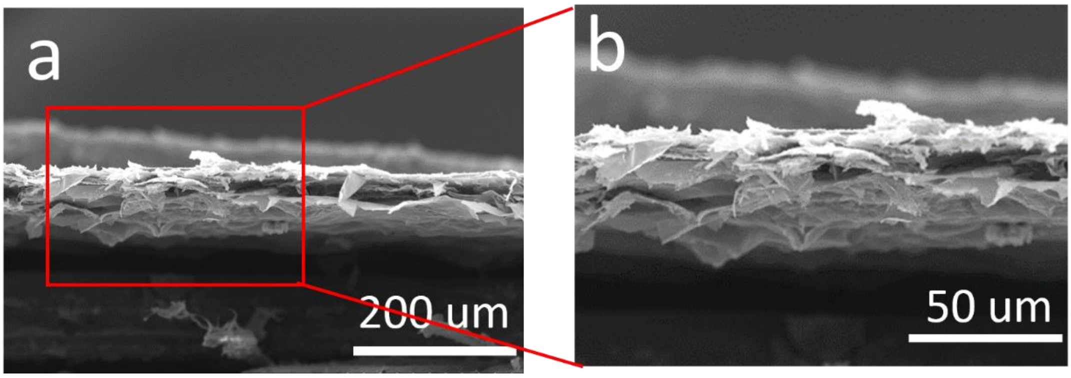

Supplementary Fig. 6 Cross-section SEM images of FS-COM-1-VF.

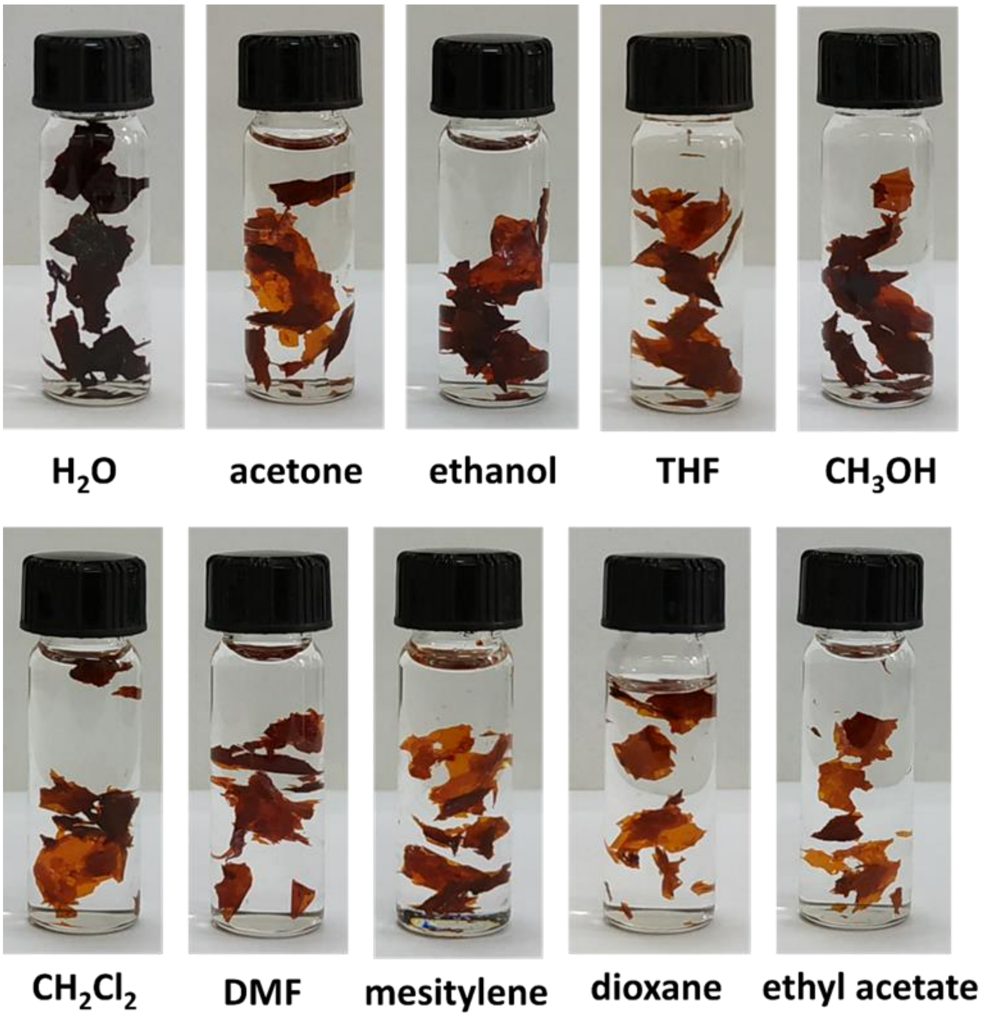

Supplementary Fig. 7 The color of COF nanosheets peeled from FS-COM-2 in different solvents.

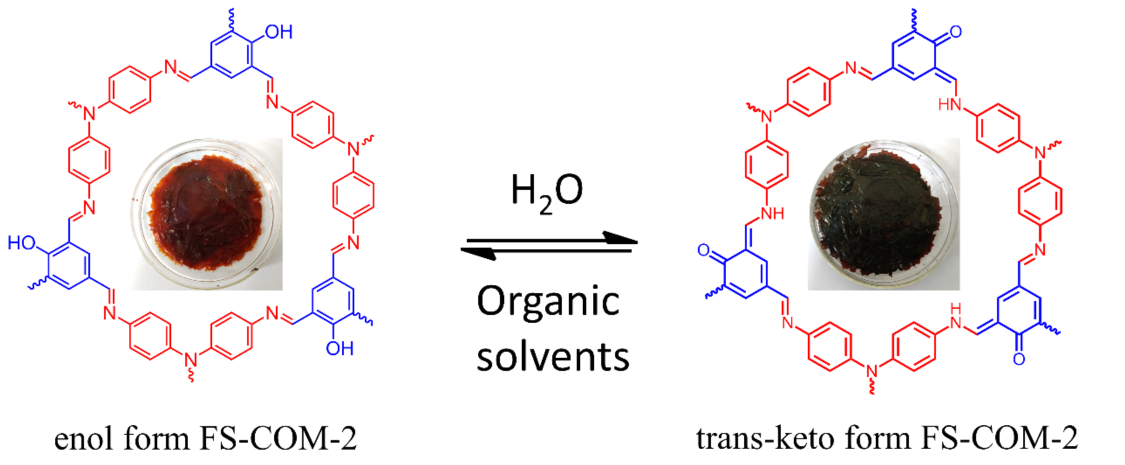

Supplementary Fig. 8 Proposed mechanism of the solvent-triggered chromism of FS-COM-2.

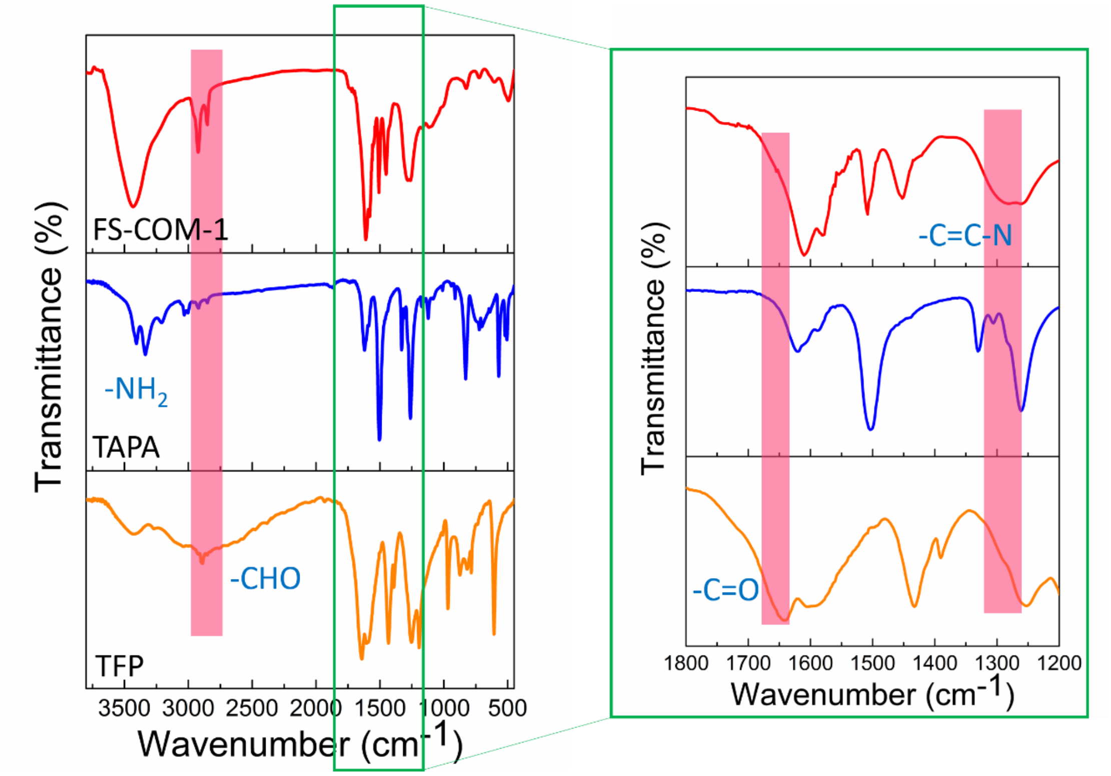

Supplementary Fig. 9 FT-IR spectra of FS-COM-1, TAPA and TFP.

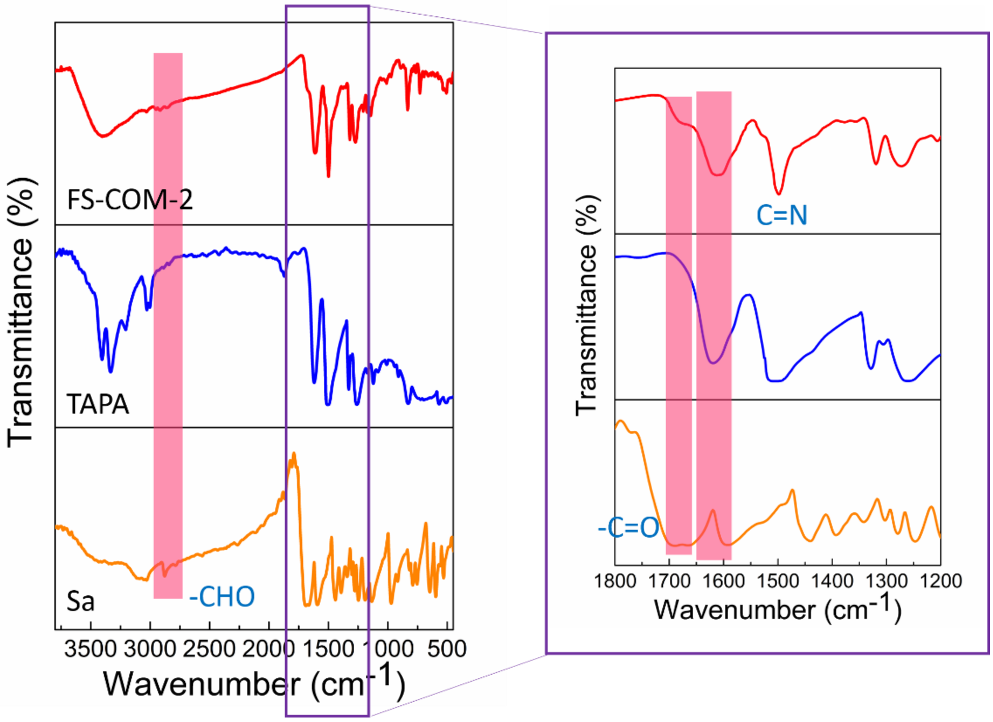

Supplementary Fig. 10 FT-IR spectra of FS-COM-2, TAPA and Sa.

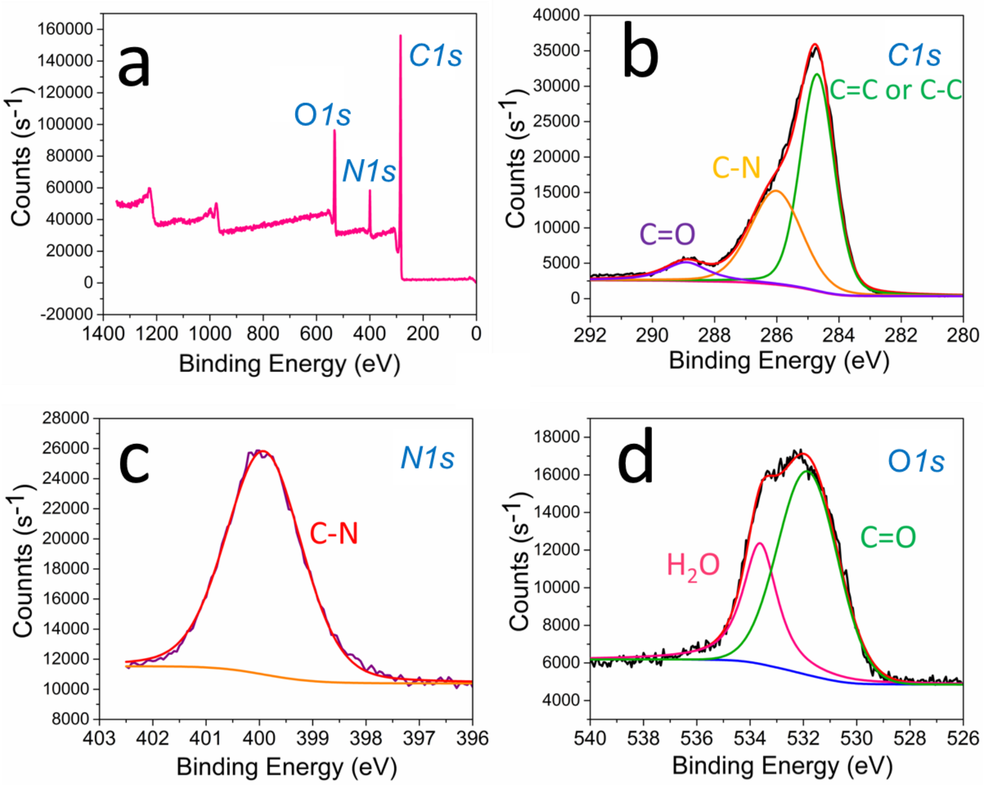

Supplementary Fig. 11 The typical XPS survey spectra (a) and high resolution XPS spectra of C*1s* (b), N*1s* (c) and O*1s* (d) of FS-COM-1.

Supplementary Table 1. Semiquantitative analysis of C/N/O content of FS-COM-1 given by XPS data.

	C atomic %	N atomic %	O atomic %
Exp.	74.47	9.53	16.00
Anal. Calcd.	79.41	11.76	8.83

Supplementary Table 2. Elemental Analysis of FS-COM-1.

	C wt %	N wt %	H wt %
Exp.	63.65	11.32	4.35
Anal. Calcd.	72.31	12.49	4.49

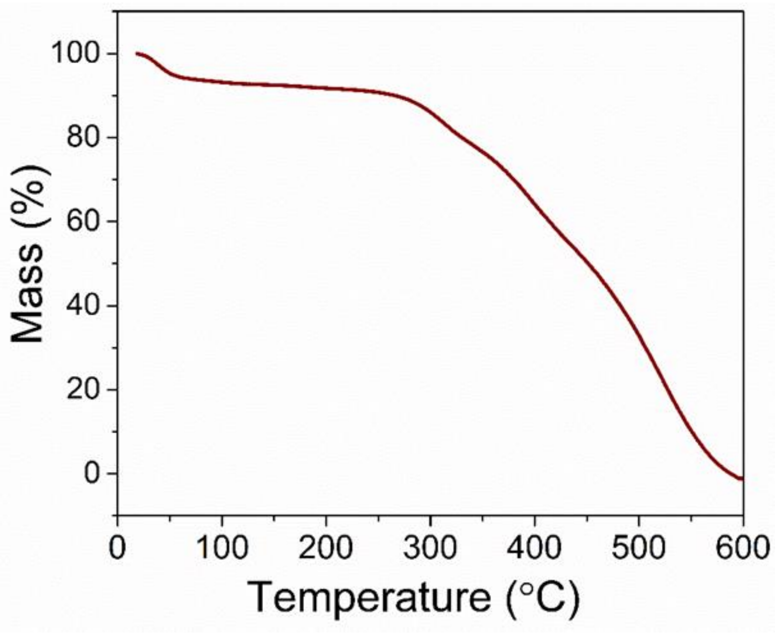

Supplementary Fig. 12 TGA curve of FS-COM-1.

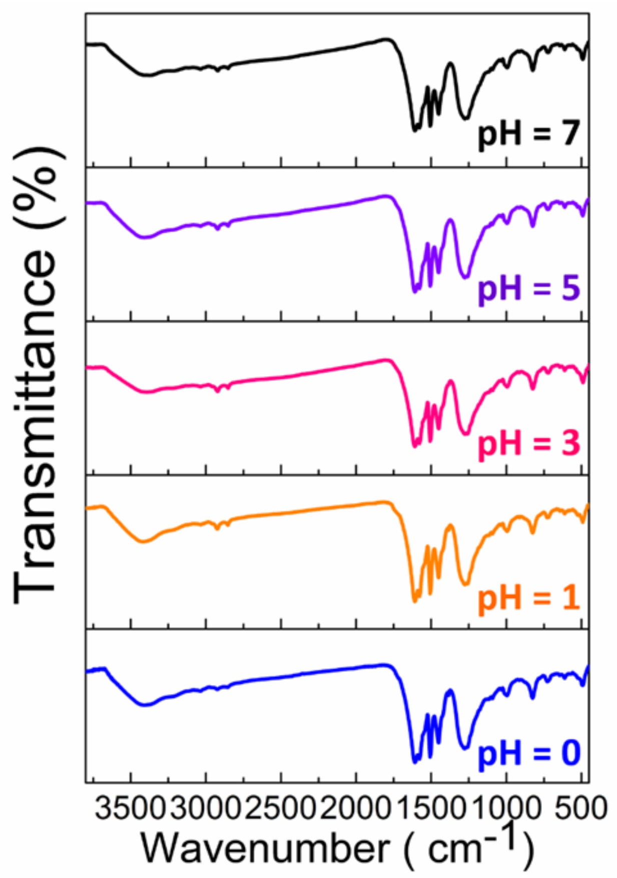

Supplementary Fig. 13 FT-IR spectra for acid stability of FS-COM-1.

Supplementary Table 3. Elemental Analysis of FS-COM-2.

	C wt %	N wt %	H wt %
Exp.	75.74	9.77	5.32
Anal. Calcd.	77.87	13.45	4.84

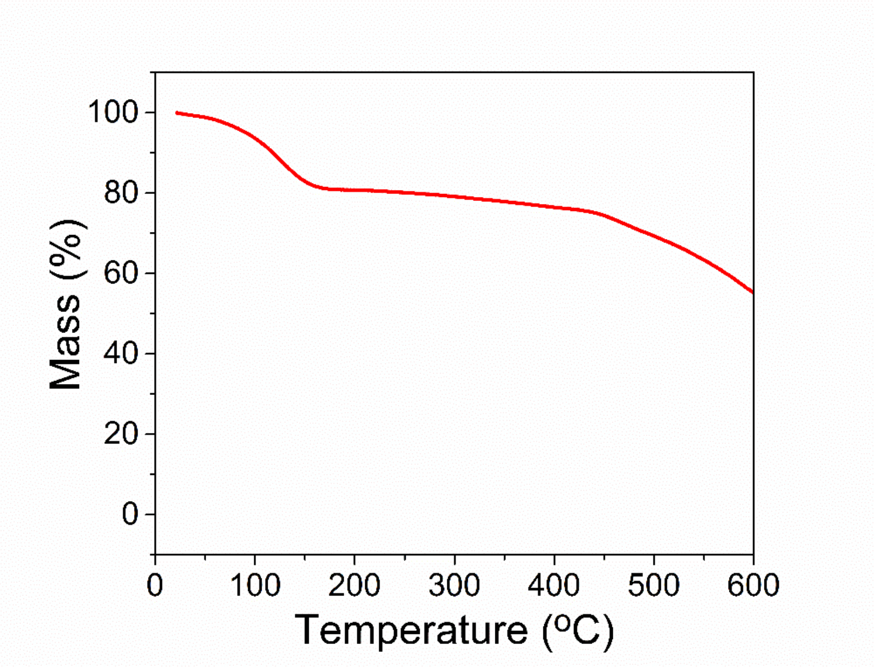

Supplementary Fig. 14 TGA curve of FS-COM-2.

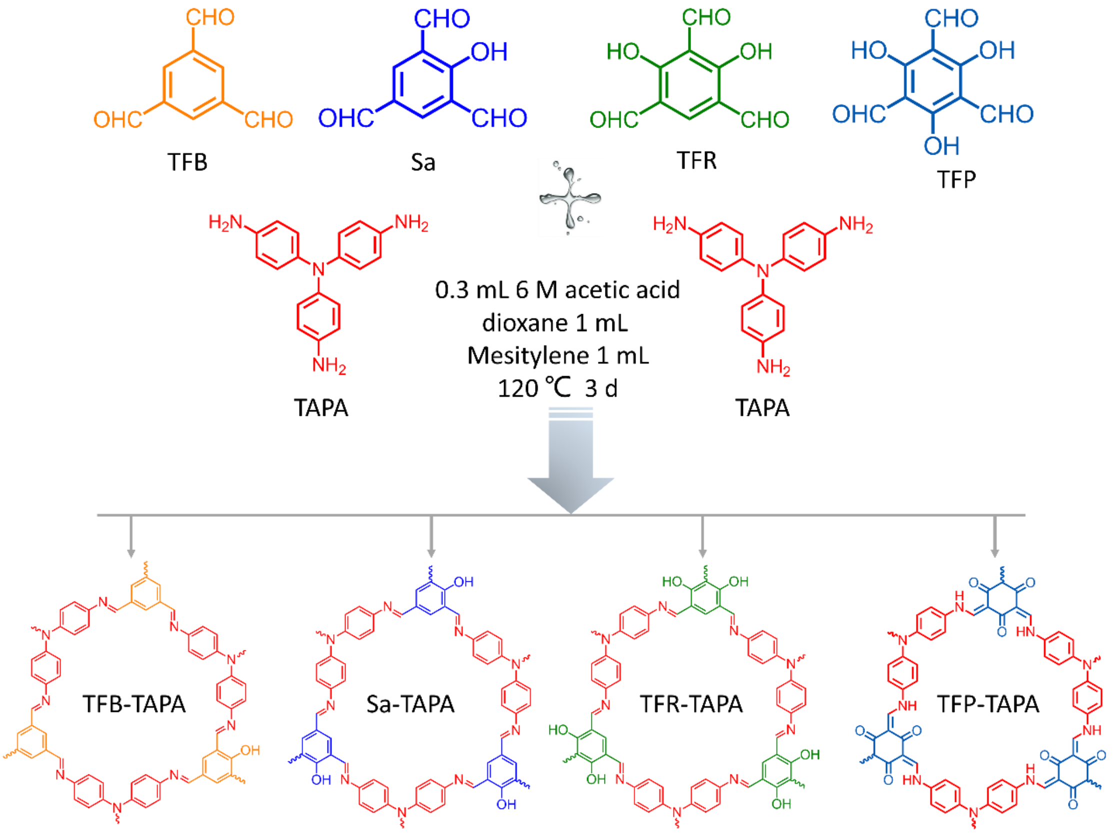

Supplementary Fig. 15 Structures and synthesis conditions of four COFs prepared by solvothermal method.

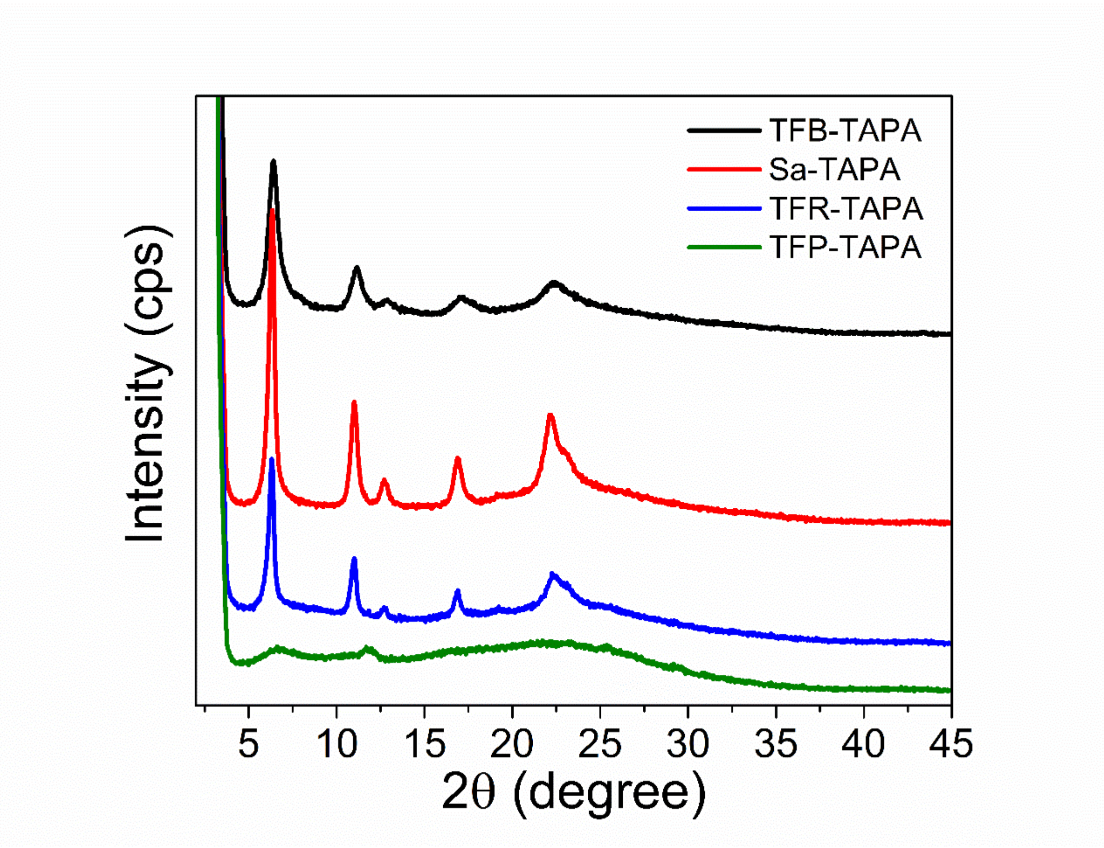

Supplementary Fig. 16 PXRD patterns of four COFs prepared by solvothermal method.

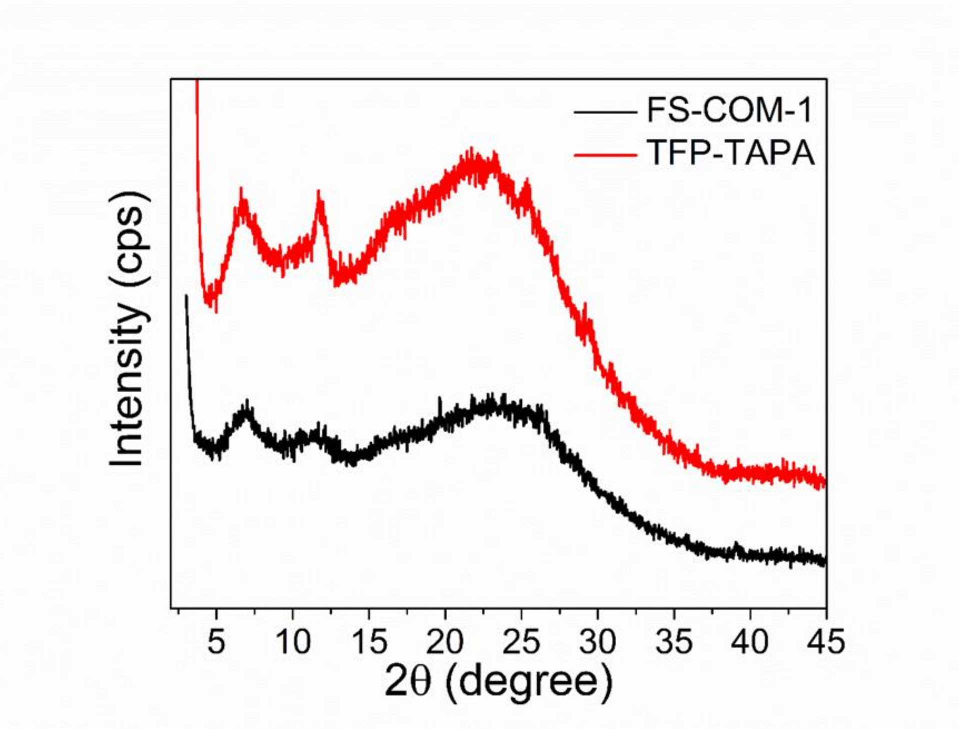

Supplementary Fig. 17 Comparison of PXRD patterns of interfacial polymerization method (FS-COM-1) and solvothermal method (TFP-TAPA).

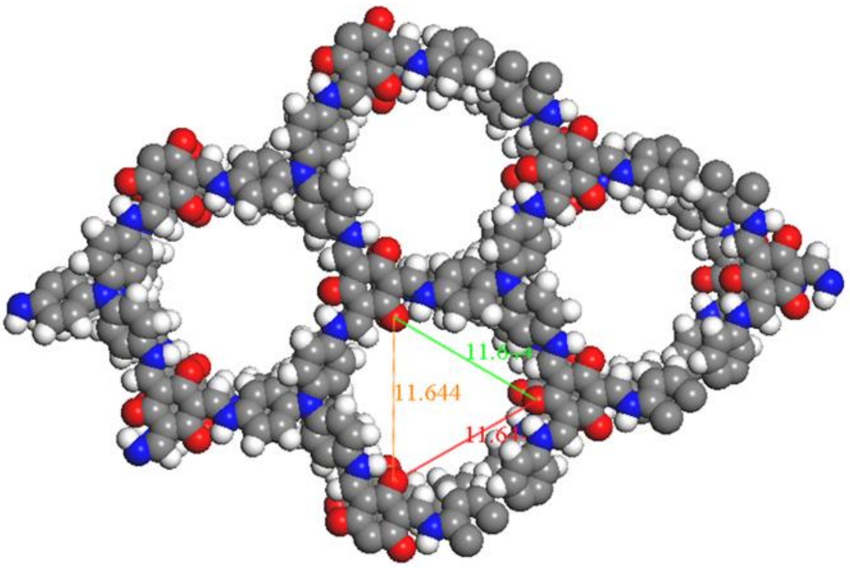

Supplementary Fig. 18 Pore size predicted by AA stacking structure of FS-COM-1.

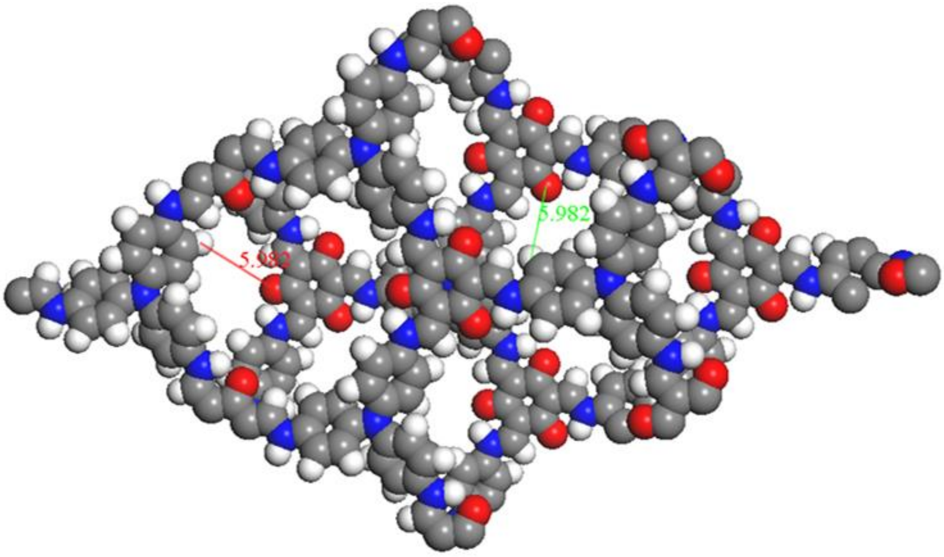

Supplementary Fig. 19 Pore size predicted by AB stacking structure of FS-COM-1.

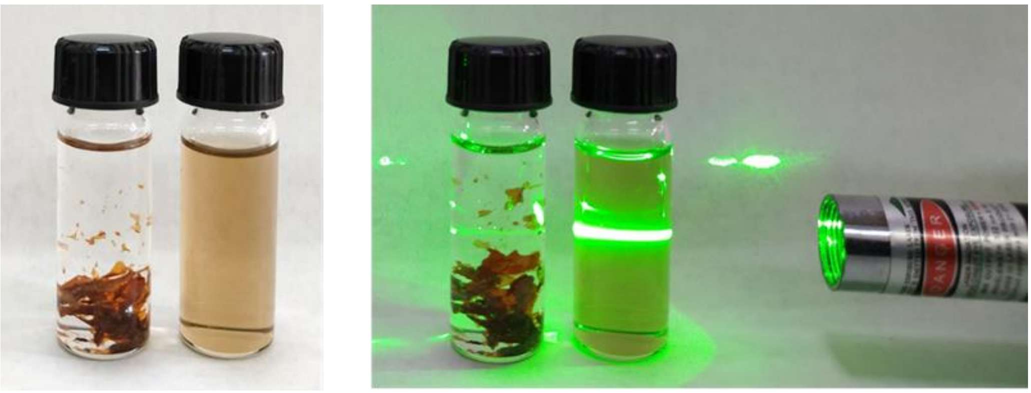

Supplementary Fig. 20 Digital photos of nanosheets and quantum dots of FS-COM-2.

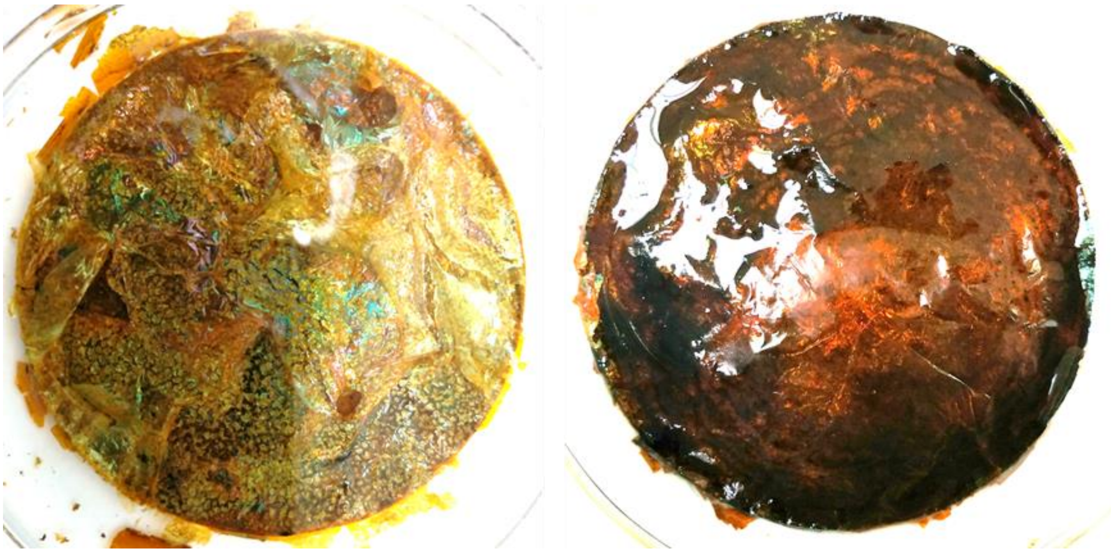

Supplementary Fig. 21 Digital photos of upper (left) and lower (right) surfaces of the FS-COM-1 membrane grown under 9M acetic acid.

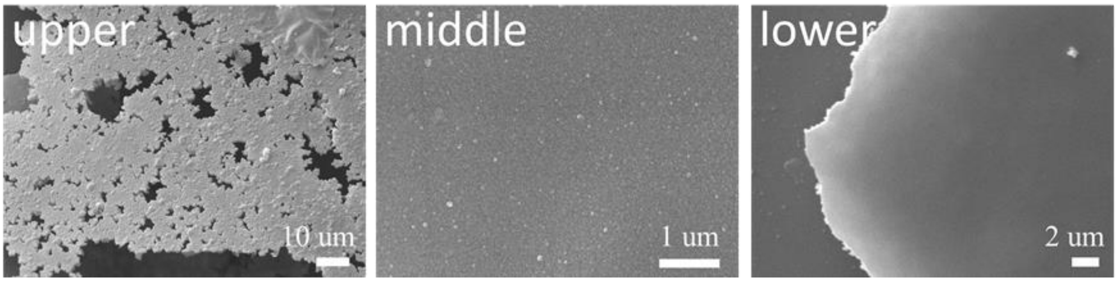

Supplementary Fig. 22 SEM images of the upper, middle and lower parts of the membrane after ultrasonic treatment.

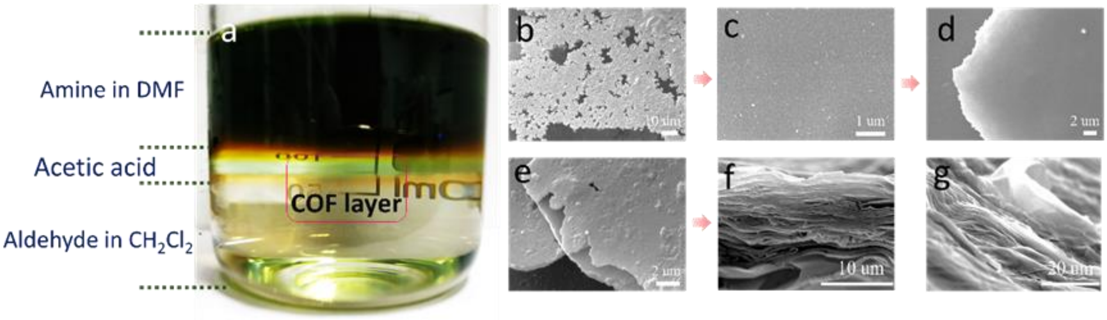

Supplementary Fig. 23 (a) The optical image of the reaction system; (b-g) SEM images of COF nanosheets at different stages.

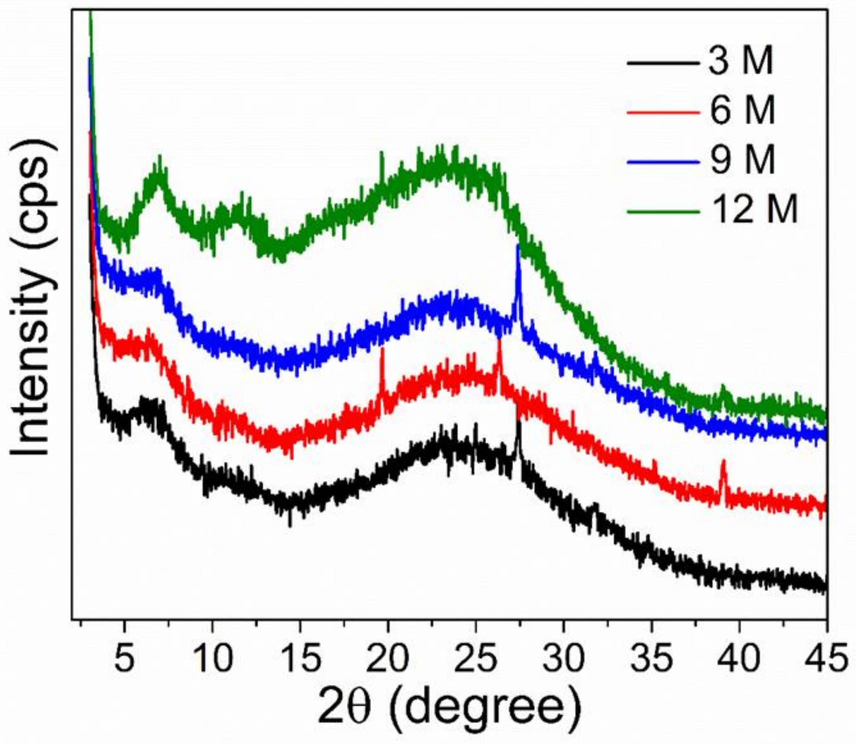

Supplementary Fig. 24 PXRD of FS-COM-1 obtained at different acetic acid concentrations.

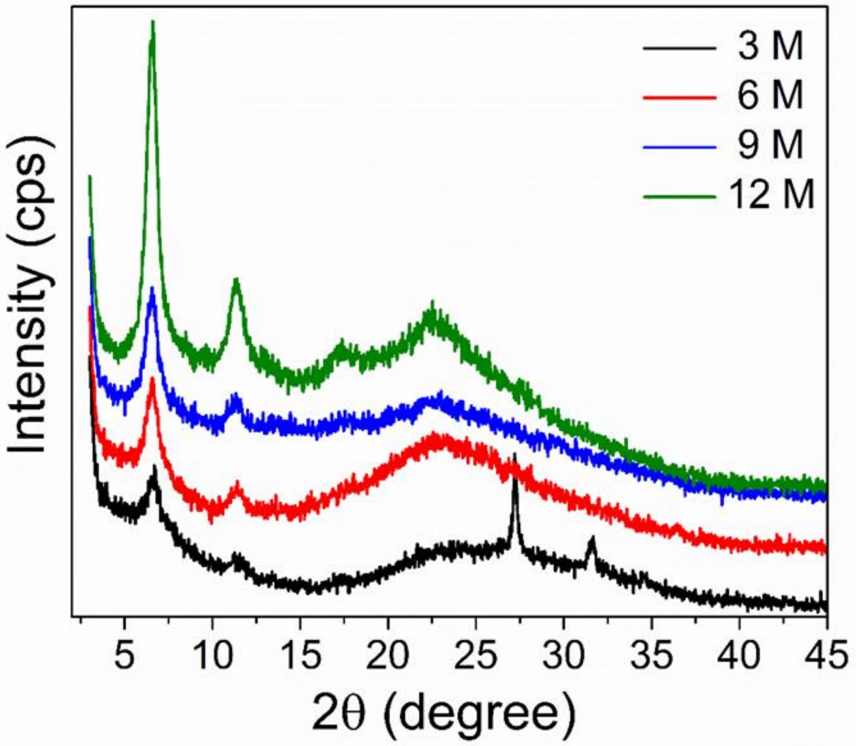

Supplementary Fig. 25 PXRD of FS-COM-2 obtained at different acetic acid concentrations.

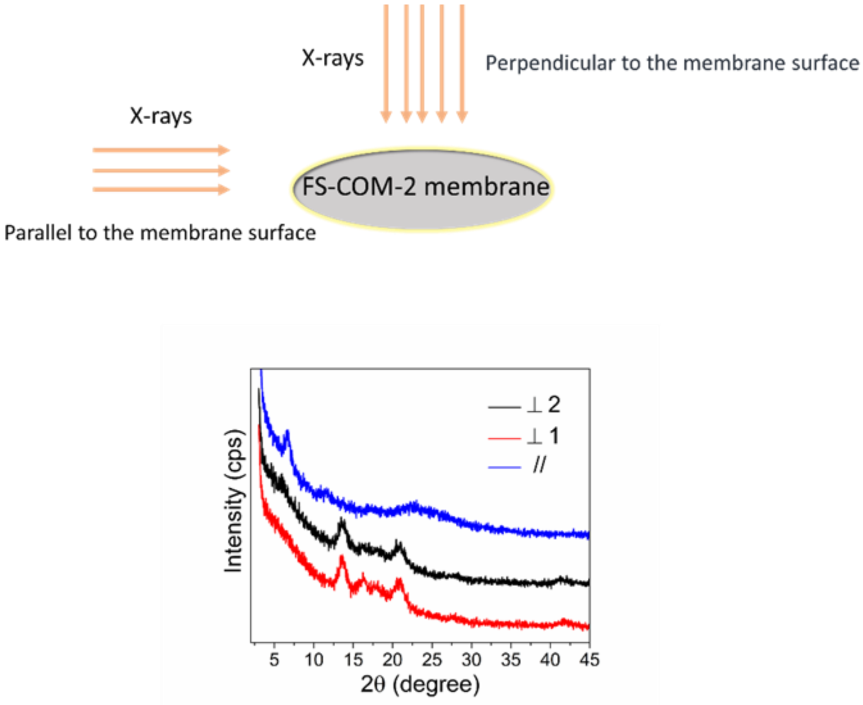

Supplementary Fig. 26 PXRD of FS-COM-2 membrane obtained at parallel and perpendicular to the membrane surface.

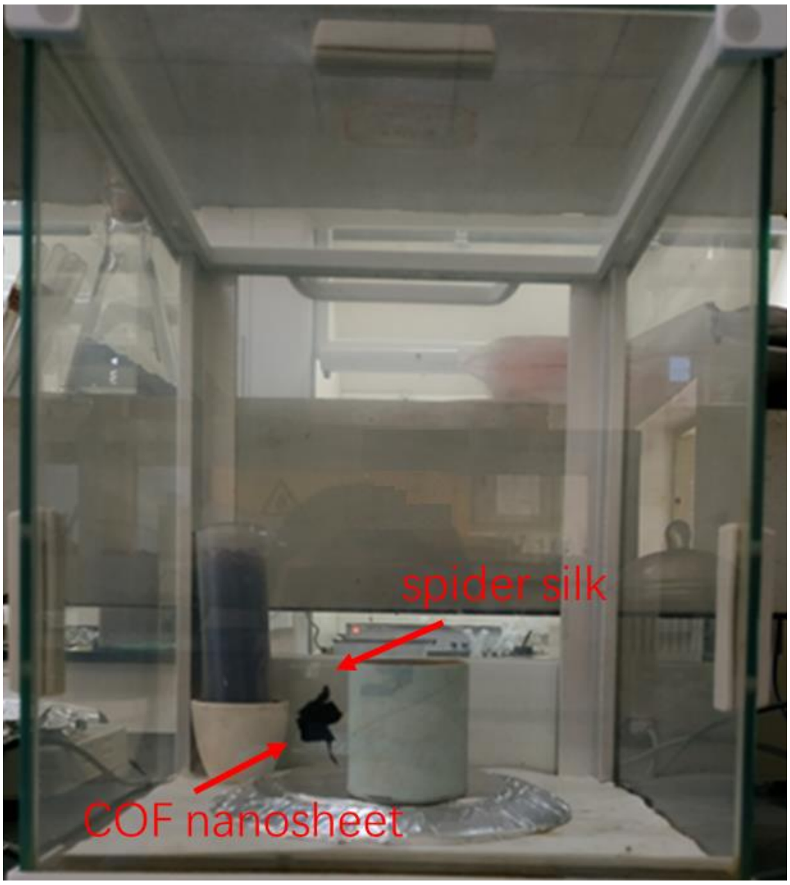

Supplementary Fig. 27 The light-driven experiment which was carried out in a closed balance and COF nanosheet and capillary tube were linked by spider silk in the experiment.

Filtration or gas separation experiments

Metal ion permeation experiments were conducted using a self-made U-shaped glass filter (diameter of 33.0 mm), which was divided into two parts, the feed part and permeate part, by the tested membrane (Supplementary Fig. 28).[3] For the permeation experiments, the feed part was equipped with 500 mL of a certain concentration of HNO_3 solution (pH = 1.8) which contained 0.5 mmol of multivalent coexisting cations, and the permeate part was installed with 500 mL of the same concentration HNO_3 solution to balance the osmotic pressure. Magnetic stirring was used in both parts to promote the permeation of cations. The cation concentration of the permeate part was measured every certain hour during the filtration experiment by ICP-OES. Dye and hydrogen ion permeation experiments were conducted by the similar steps. However, it is difficult to measure water permeation flux through the above procedures. To do this, we further designed and manufactured a pressure filtration apparatus (Supplementary Fig. 32 and S33) for pressure filtration test. We studied the permeance of common solvents for FS-COM-1 under a transmembrane pressure of 0.1 MPa. The cation concentration of the permeate part was measured by ICP-OES and the volumes of solvents were measured by a measuring cylinder.

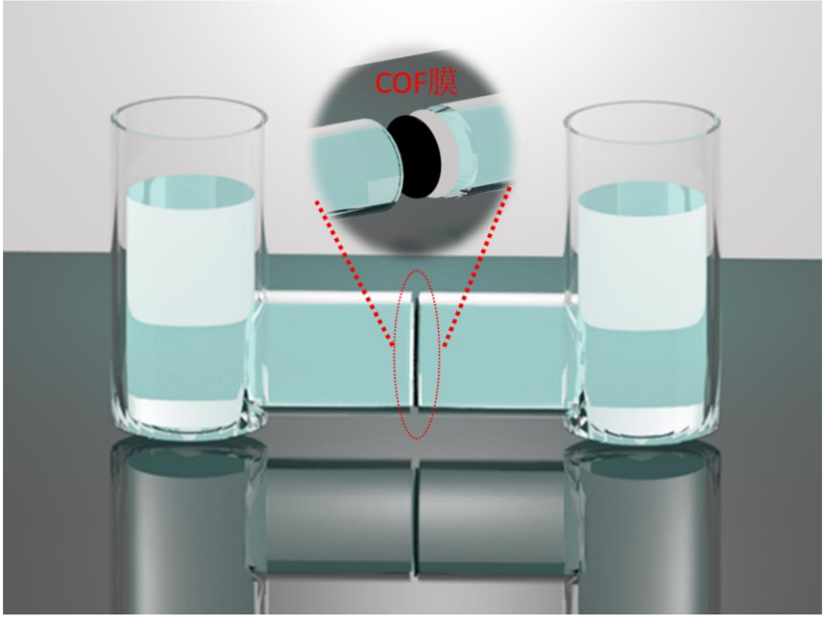

Supplementary Fig. 28 Diagram of the U-shaped glass filter used in permeation experiments. Feed part (left), permeate part (right), FS-COM membrane is fixed at the joint between the two filter bowls.

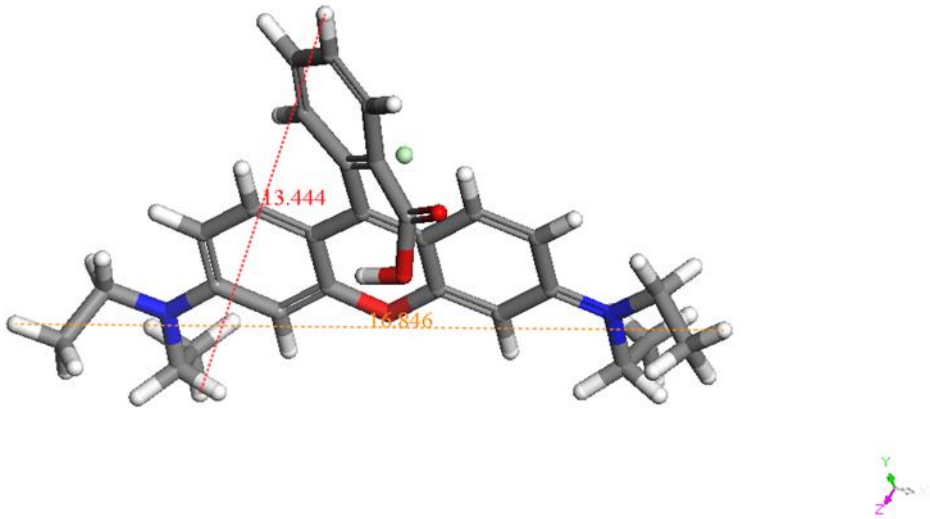

Supplementary Fig. 29 Molecule size of rhodamine B.

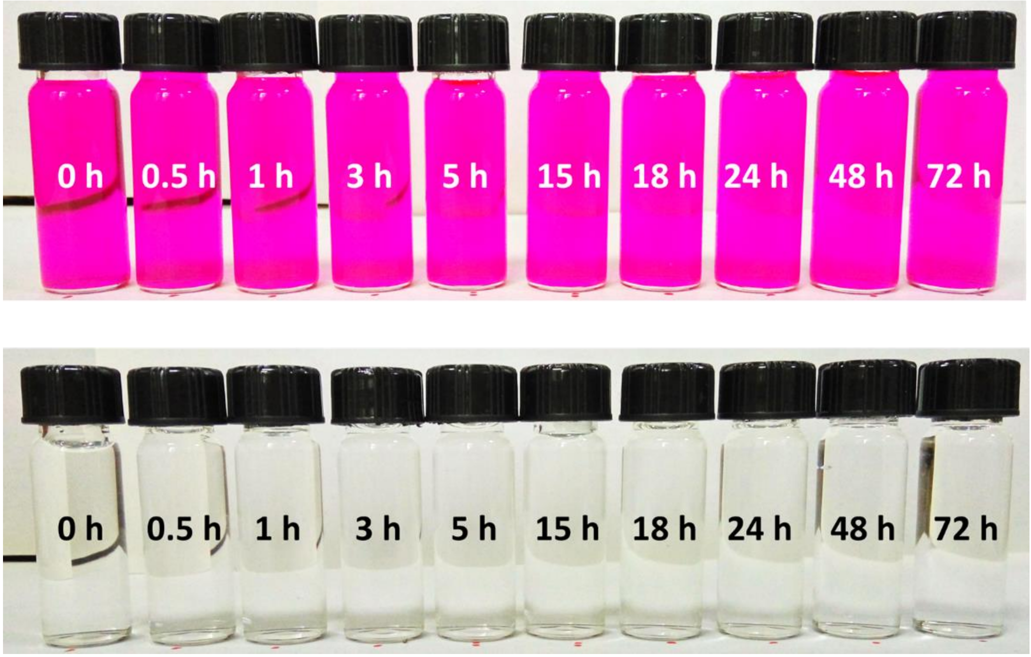

Supplementary Fig. 30 Digital photographs of rhodamine B aqueous solution before and after filtration through FS-COM-1 at different times.

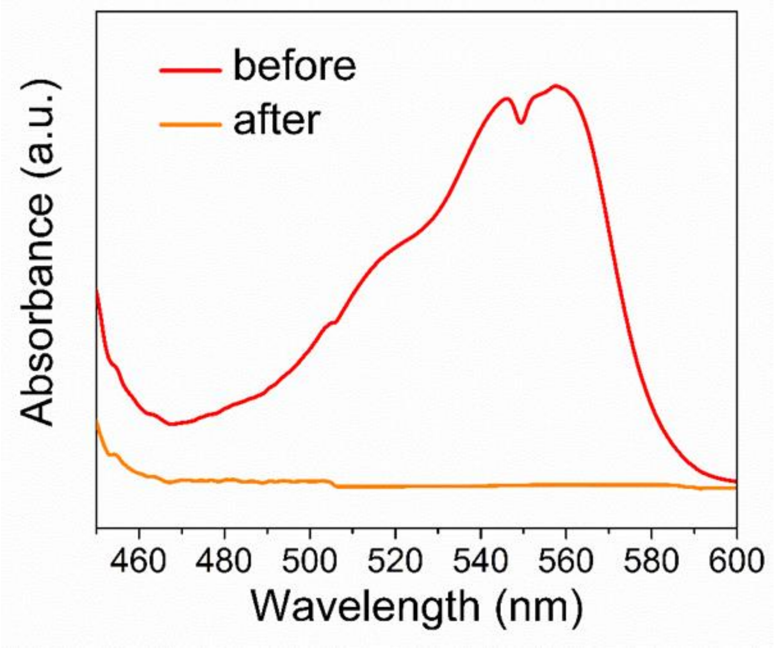

Supplementary Fig. 31 UV−vis absorption spectra of rhodamine B before and after permeation through FS-COM-1.

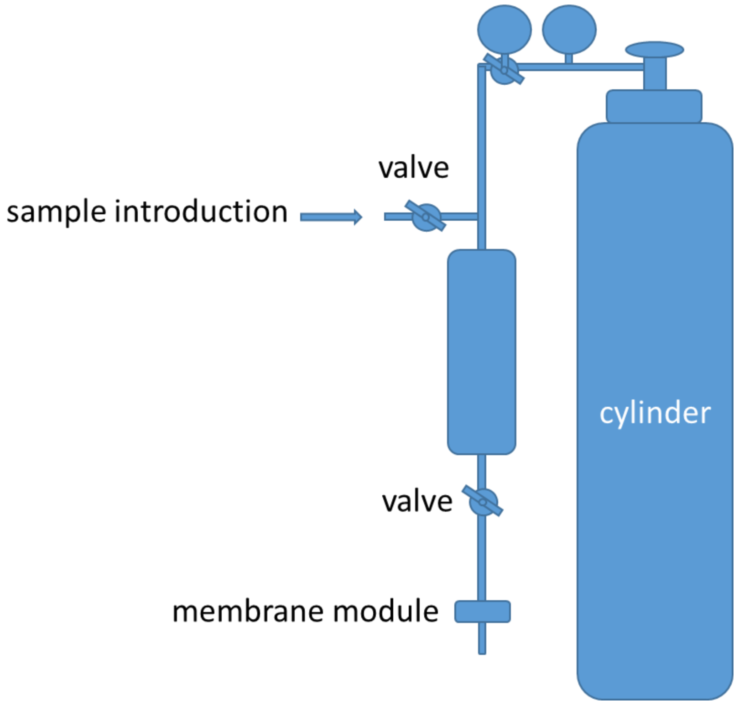

Supplementary Fig. 32 The pressure filtration apparatus used in the experiment.

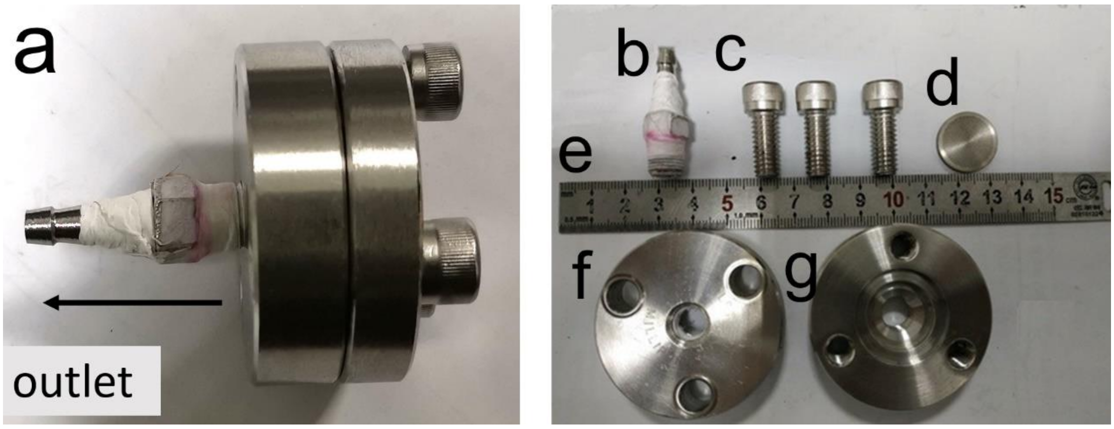

Supplementary Fig. 33 Membrane module device (a) and its components. (b) nozzle; (c) hexagonal nuts; (d) grid for supporting COF membranes; (e) steel ruler for size measurement; (f,g) flanges.

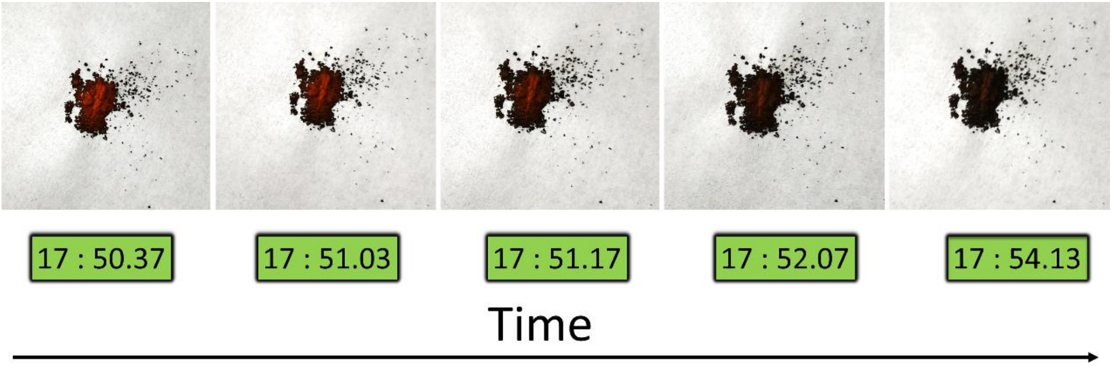

Supplementary Fig. 34 Color changes of FS-COM-2 exposure to the air. The number in the green box is the photo taken time.

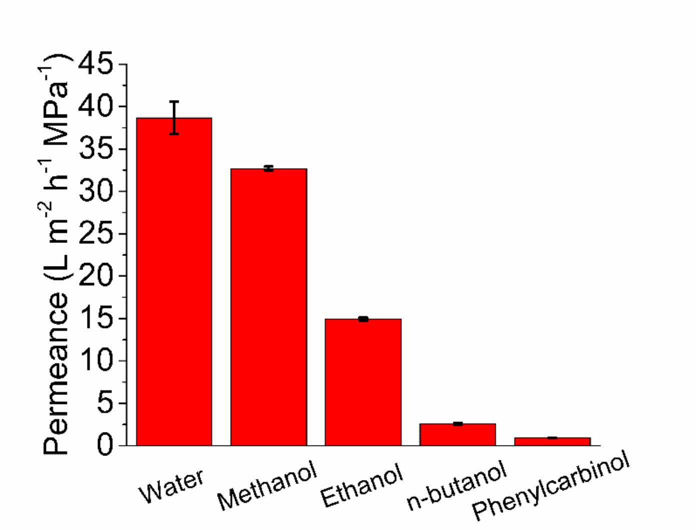

Supplementary Fig. 35 Permeance of solvents with increasing size for FS-COM-1.

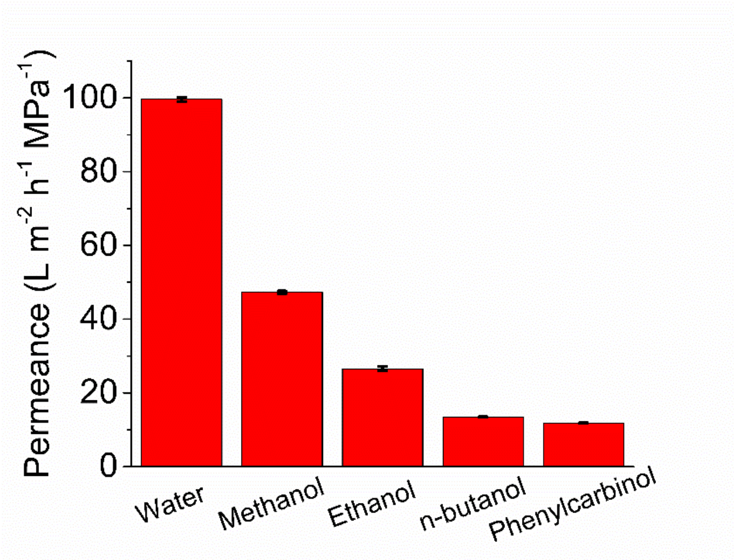

Supplementary Fig. 36 Permeance of solvents with increasing size for FS-COM-2.

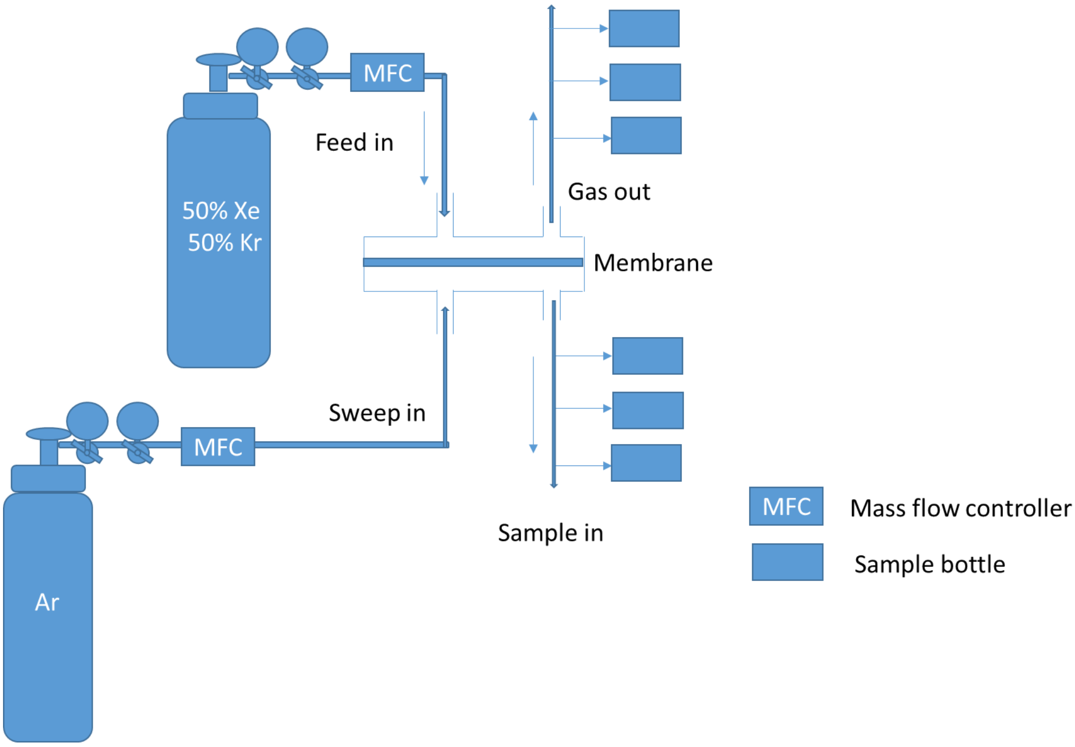

Supplementary Fig. 37 Illustration of gas separation measurement apparatus (Wicke-Kallenbach type).

Gas separation tests of the FS-COM-1.

The obtained membranes were sealed in Wicke-Kallenbach cells to measure their separation performance for Xe/Kr gas mixture (see Supplementary Fig. 37). The mixed feed (Xe/Kr 1:1 mixtures) flow rates were constant with a total volumetric flow rate of 200 mL/min, with 100 mL/min of each gas (1:1 mixture), regulated by mass flow controllers (MFCs). Argon, a relatively bulky molecule, was used as a sweep gas to minimize the influence of backdiffusion of the sweeping gas to the feed side. There was no pressure drop between the sides of the membranes, in order to prevent any distortion of FS-COM-1. The separation factor is defined as the molar ratio of Kr to Xe in the permeate over the molar ratio in the feed. The molar fraction of each component gas can be determined by mass spectrum. Gas permeation measurements were conducted at the following conditions:

atmospheric pressure = 97000 Pa, experimental temperature = 25℃.

Gas permeance is calculated by the following equation:

$$P_i = \frac{N_i}{A \Delta p_i} \quad (1)$$

$$S_{i/j} = \frac{y_i^F / y_j^F}{y_i^S / y_j^S} \quad (2)$$

where P_i is the permeance of component i (mol m^{-2} s^{-1} Pa^{-1}), N_i is the permeate rate of component i (mol s^{-1}), A is the effective membrane area (m^2), and $\triangle P_i$ is the transmembrane pressure difference of component i (Pa).

Supplementary Table 4. Xe/Kr separation performance of FS-COM-1.

Sample	Kr%	Xe%	P_{Kr}	P_{Xe}	$S_{Xe/Kr}$
			× 10^{-8} mol m^{-2} s^{-1} Pa^{-1}		
1	0.121	0.109	1.286	1.158	1.11
2	0.119	0.107	1.265	1.137	1.11
3	0.121	0.108	1.286	1.148	1.12

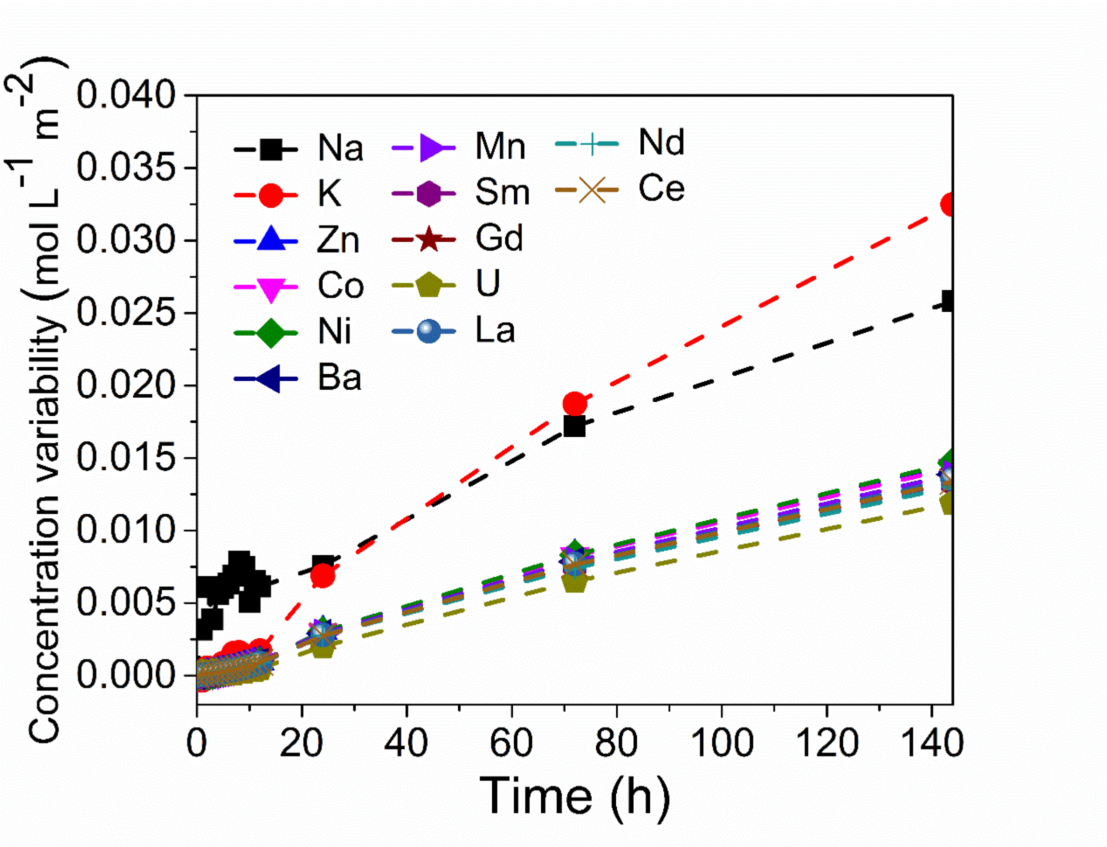

Supplementary Fig. 38 Concentration variability of 14 coexisting cations in the permeate part for FS-COM-2.

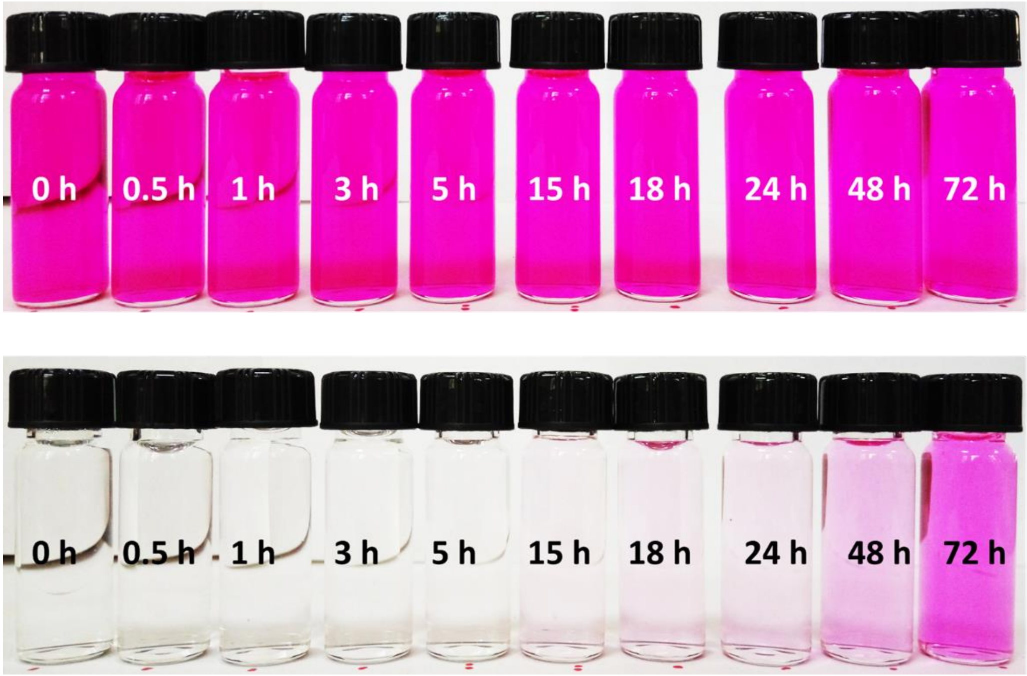

Supplementary Fig. 39 Digital photographs of rhodamine B aqueous solution before and after filtration through FS-COM-1-VF at different times.

Supplementary References

1. Chong, J. H.; Sauer, M.; Patrick, B. O.; MacLachlan, M. J. Org. Lett. 2003, 5, 3823.
2. Li, Y.; Zhang, M.; Guo, X.; Wen, R.; Li, X.; Li, X.; Li, S.; Ma, L. Nanoscale Horiz., 2018, 3, 205.
3. Wang, L.; Guo, X.; Cao, K.; Li, B.; Li, Y.; Zhang, M.; Wen, R.; Li, X.; Li, S.; Ma, L. J. Mater. Chem. A, 2017, 5, 8051.
