# Supplementary material for: Laminated self-standing covalent organic framework membrane with uniformly distributed subnanopores for ionic and molecular sieving
Source: Nat Commun. 2020 Jan 30;11:599. doi: 10.1038/s41467-019-14056-7 (PMC6992836; doi:10.1038/s41467-019-14056-7)
Supplement: Supplementary file 2 — Description of Additional Supplementary Files [file 41467_2019_14056_MOESM2_ESM.pdf]

Description of Additional Supplementary Files

File Name: Supplementary Movie 1

Description: Supplementary Movie 1 for the peeling process. In Supplementary Movie 1, we use a spoon to peel off the laminated, pliable, free-standing COF membrane (FS-COM-1) collected from the organic-organic interface. What we want to express in the video is that the layer-by-layer nanosheets can be peeled off easily, even with a spoon.

File Name: Supplementary Movie 2

Description: Supplementary Movie 2 for the light-driven progress. In Supplementary Movie 2, what we want to express in the video is the low density of the membrane. We tried to hit the COF membrane (~ 0.7 mg) with a laser and to see if it could be driven (The power of the laser pointer is 3 mW). To prevent the influence of air flow, the experiment was carried out in a closed balance. And in the experiment, COF nanosheet and capillary tube were linked by spider silk. Delightedly, the COF membrane can do be driven by the red laser. A video about the light-driven process is attached in the Supplementary Movie 2.
